# Comparison of Executive Function Skills between Patients with Cerebral Palsy and Typically Developing Populations: A Systematic Review and Meta-Analysis

**DOI:** 10.3390/jcm13071867

**Published:** 2024-03-24

**Authors:** Nóra Zimonyi, Tamás Kói, Viktor Dombrádi, Marcell Imrei, Rita Nagy, Márk Ágoston Pulay, Zsolt Lang, Péter Hegyi, Zsofia K. Takacs, Ibolya Túri

**Affiliations:** 1 Centre for Translational Medicine, Semmelweis University, 1085 Budapest, Hungary; samatiok@gmail.com (T.K.); marcell.imrei@gmail.com (M.I.); nagyrita003@gmail.com (R.N.); markpulay@gmail.com (M.Á.P.); lang.zsolt@univet.hu (Z.L.); hegyi2009@gmail.com (P.H.); 2 Pető András Faculty, Semmelweis University, 1085 Budapest, Hungary; turi.ibolya@semmelweis.hu; 3 School of PhD Studies, Semmelweis University, 1085 Budapest, Hungary; 4 Department of Stochastics, Institute of Mathematics, Budapest University of Technology and Economics, 1111 Budapest, Hungary; 5 Patient Safety Department, Health Services Management Training Centre, Faculty of Health and Public Administration, Semmelweis University, 1085 Budapest, Hungary; dombradi.viktor@emk.semmelweis.hu; 6 Institute for Translational Medicine, Medical School, University of Pécs, 7622 Pécs, Hungary; 7 Heim Pál National Pediatric Institute, 1089 Budapest, Hungary; 8 Department of Ergonomics and Psychology, Faculty of Economic and Social Sciences, Budapest University of Technology and Economics, 1111 Budapest, Hungary; 9 Department of Biostatistics, University of Veterinary Medicine Budapest, 1078 Budapest, Hungary; 10 Institute of Pancreatic Diseases, Semmelweis University, 1085 Budapest, Hungary; 11 School of Health in Social Science, University of Edinburgh, Edinburgh EH10 5HF, UK; zgarait@ed.ac.uk

**Keywords:** cerebral palsy, executive functions, working memory, inhibitory control, cognitive flexibility

## Abstract

**Background**: Children with CP show deficits in executive function compared to their typically developing peers, based on the majority of the available evidence. However, the magnitude of these deficits, as well as the proportions of the shortfalls in the three main components, have not yet been examined. This is the first meta-analysis to synthesize evidence on the magnitude of differences between patients with cerebral palsy (CP) and typically developing populations in different components of executive function skills (working memory, inhibitory control and cognitive flexibility), and thus makes recommendations on which areas of executive functioning are in greatest need of intervention. **Methods**: We conducted a systematic literature search of four databases for studies that measured executive functions in these two groups until 31 August 2023. We calculated the standardized mean difference (Hedges’ g), an average effect size overall, and for the three components of executive function skills separately, we used several moderator analyses, including methodological differences between the primary studies. **Results**: Fifteen articles were included in the meta-analysis. The average mean difference in executive functioning overall was large (g+ = −0.82). Furthermore, large significant differences were found in working memory (g+ = −0.92) and inhibitory control (g+ = −0.82) and a moderate difference was identified in cognitive flexibility (g+ = −0.57). In addition, results of moderator analyses reveal the importance of a rigorous matching of control group participants and CP patients. **Conclusions**: The results demonstrate a severe impairment in all executive functions among CP patients compared to typically developing peers, which do not decrease over time.

## 1. Introduction

Cerebral palsy (CP) is the most common motor and movement disability in childhood with an incidence of 2–3 cases per 1000 live births [1,2]. In addition to the issue of gross motor skills, various motor abilities are also affected in the case of CP. The definition of CP was updated in 2006 to recognize that ‘the motor impairments of CP are frequently accompanied by sensory, perceptual, cognitive, communication, and behavioral disturbances, as well as epilepsy, and secondary musculoskeletal issues’ [3]. CP is a heterogeneous neurodevelopmental disorder, and there are two main classification systems to assess its severity. The gross motor impairment (e.g., sitting and walking) of patients with CP is often graded using the Gross Motor Function Classification System (GMFCS), which ranges from 1 to 5, with 5 representing the most severe category, and The Manual Ability Classification System (MACS) is widely utilized to assess hand manipulation in individuals with CP, consisting of 5 levels. A higher level on the scale indicates a more severe condition [4].

Although research on the executive functions of the CP population is ongoing, many questions still remain. Executive functions (EF) refer to the cognitive aspect of self-regulation, comprising a range of interrelated skills required to perform goal-directed, non-automatic behaviors. These domain-general skills are important for several cognitive, emotional, and social outcomes such as academic functioning [5,6,7,8] and social-emotional wellbeing [9,10]. Longitudinal research indicates that early life deficits in executive functioning can contribute to the emergence of behavioral problems later in life [11].

EF is generally considered to consist of three related but separable components [12]. (1) Updating or working memory allows us to store and manipulate information in our minds temporarily. (2) Inhibition includes self-control, inhibition of unwanted behavior, selective attention and cognitive control. (3) Cognitive flexibility or shifting refers to the ability to approach a problem from different perspectives and to shift flexibly between mental sets. These three components are assessed using various neuropsychological tests, for example, the Stroop, Digit Span, Corsi Block-Tapping and Wisconsin Card Sorting Tests.

Although, there is not yet a substantial body of literature on the topic, the majority of available evidence suggests that children with CP have a deficit in executive functions compared to their typically developing peers, which has been confirmed in previous literature reviews [13,14,15,16]. However, the magnitude of this deficit and whether it varies across components remains unexplored; to our knowledge, the present meta-analysis is the first that investigated the three components separately.

Executive function skills, associated with prefrontal brain regions, develop throughout childhood, adolescence and even early adulthood [17]. Thus, it is unclear whether children with CP experiencing deficits in EF skills will catch up with their typically developing peers by the end of this developmental process or whether the deficit remains in adulthood [13]. 

Taking these into consideration, our aim was to synthesize the evidence on the magnitude of differences between patients with CP and typically developing populations in the different components of executive function skills and thus make recommendations on which areas of executive functioning are in greatest need of intervention. Furthermore, another objective of the present meta-analysis was whether functional impairments represented only a developmental delay or a permanent impairment in the CP population.

We hypothesized that the CP group would show a deficit on executive function tests. Beyond that, we did not have hypotheses regarding differences between different components and whether the deficit represents a developmental delay or a permanent impairment due to the lack of evidence in the field.

## 2. Materials and Methods

The meta-analysis is reported according to the Preferred Reporting Items for the Systematic Reviews and Meta-Analyses (PRISMA) statement [18]. The protocol of this study was pre-registered on the International Prospective Register of Systematic Reviews (PROSPERO) website under registration number CRD42021292221. There were no deviations from the protocol. The operational definitions of EF components can be found in the S1 Supplementary Material.

### 2.1. Data Sources and Search Strategy

On 31 August 2023, we ran a systematic literature search in four literature databases: MEDLINE (via PubMed), Embase, Cochrane Register of Controlled Trials (CENTRAL), and Web of Science. 

We designed a search key containing terms associated with executive functions and cerebral palsy that can be found in the S2 Supplementary Material. We did not employ a specific publication time interval during the study search.

Furthermore, a manual search for any additional studies was performed in both cited and citing papers (via Google Scholar) of the included studies and relevant reviews. All disagreements were resolved by consensus. When it was necessary, we contacted the authors of the primary studies for missing data.

### 2.2. Selection and Eligibility

The selection process was based on the title, abstract, and full text by two independent reviewers (NZ and MP) using the following inclusion criteria: only cross-sectional studies containing data comparing CP and a typically developing population; results that reported on at least one measure of executive function skills, either measured by neurocognitive tests or reported by the parent or teacher; finally, the paper was written in English.

Studies were excluded that reported on samples in which cerebral palsy developed later in life (e.g., as a result of a stroke). Studies comparing a group with CP to another disorder were also excluded.

We included any measure of executive function skills including working memory, inhibitory control, cognitive flexibility and planning. We excluded measures of short-term memory, e.g., a span test in which participants were only required to recall items in the same order as presented. We considered span tests in which participants were instructed to recall items in the opposite order as measures of working memory; these were subsequently included in the meta-analysis, as working memory is a capability to retain and manipulate information in the mind [19]. We coded only data on the accuracy of neurocognitive tests and excluded results on reaction times to ensure that the motor speed in CP patients did not influence the results. Cohen’s kappa coefficients were used to determine the level of agreement between reviewers. All disagreements were resolved by consensus.

### 2.3. Data Extraction 

Two researchers coded every article according to a standardized coding schema (NZ and DK). Interrater reliability ranged from 80% to 100%, which reflects good reliability. Disagreements were resolved by a third researcher (ZKT).

The following data were extracted from each eligible article: bibliographic information, country where the data was collected, the specific diagnosis of the CP group, sex and age distribution, number of participants, whether groups were matched through demographic characteristics (e.g., age, sex), scores of CP groups on the GMFCS and the MACS, different measures of every executive function skills that could be included and whether it was a neurocognitive test or a questionnaire, in addition to the component(s) tested (e.g., working memory, inhibitory control, and cognitive flexibility), and whether it measured verbal or nonverbal working memory for the working memory tests. Mean and standard deviations (SD) were extracted for EF. 

### 2.4. Quality Assessment

We used the “Quality In Prognosis Studies” (QUIPS) risk of bias assessment tool according to the recommendations of the Cochrane Collaboration [20]. This was verified by a second researcher (ZKT). Criteria for the QUIPS domains are provided in the S3 Supplementary Material.

### 2.5. Statistical Analysis

Statistical analyses were carried out using ‘meta’, ‘metafor’ and ‘clubSandwich’ packages of the R statistical software (version 4.1.2). The statistical analyses followed the recommendations of Harrer et al. [21]. For all statistical analyses, a *p*-value of less than 0.05 was considered significant.

CP and control groups were compared by performing several tests. For each test in each study, we calculated Hedges’ g standardized mean difference along with its standard error between the patient performances in the CP and typically developing control groups. We paid special attention to the sign of the Hedges’ g-s: negative values always indicate that the performance in the CP group is worse than that of the normal performing group. In several cases, the study divided CP patients into two subpopulations. In these cases, we calculated a combined mean and SD [22]. For each study and executive function skill category, we took the averages of the calculated Hedges’ g scores of tests in the same category. We then used a conservative approach to obtain the standard errors: we upper-bounded the standard errors of the averages by the averages of the standard errors of the Hedges’ g-s of the included tests, and we used these upper-bounds in the meta-analyses.

We applied random-effects meta-analysis for the averages of the Hedges’ g differences in the three groups. We used the classical inverse variance method with the restricted maximum likelihood estimator. As not many studies contributed to the meta-analysis, Hartung–Knapp adjustment was applied. Besides the prediction interval, heterogeneity was assessed by calculating the I^2^ measure and its confidence interval and by performing the Cochrane Q test. I^2^ values of 25%, 50%, and 75% were considered low, moderate, and high heterogeneity, respectively [22].

We performed several moderator analyses by applying subgroup analysis or meta-regression. As a sensitivity analysis, we also performed baseline analyses using the methodology of Pustejovsky and Tripton [23], considering the Hedges’ g-s as correlated outcomes [24].

We assessed publication bias visually using a funnel plot. We also performed Egger’s test when at least ten studies were available.

### 2.6. Subgroup and Meta-Regression Analyses

We examined whether the difference in EF between cerebral paretic and typically developing children showed a decreasing trend with increasing age. With this analysis, we aimed to assess whether the disadvantage in executive function skills of samples with CP was more pronounced in childhood than in adulthood, suggesting a developmental delay, as opposed to a permanent deficit that persisted throughout the lifetime of patients. We conducted a meta-regression so that the mean age of the samples in the primary studies predicted the size of the effect. Additionally, due to the limitations of such an approach, we also categorized the primary studies by broad age groups of children and adults, and conducted a subgroup analysis to compare the effect sizes in these two groups. In line with the principle of continuity, we also compared the different age groups of children (early childhood 4–6 years old, childhood 7–13 years old, and adolescence 14–18 years old).

Further subgroup analyses were conducted to compare contrasts based on categorical moderator variables, such as the difference between verbal and visuospatial working memory and the difference between measures of planning and shifting within cognitive flexibility. Differences between samples with less severe conditions according to the GMFCS (<3) and MACS (<2) were compared with samples of participants of various severity. We also compared differences in the main diagnosis (spastic or dyskinetic), differentiated diagnoses within spastic (diplegia, hemiplegia (at least 80% within the group had diplegia or hemiplegia) and mix (mono-, di-, tetraplegia)). Further differences were examined between data collected in different continents, as well as whether the typically developing sample matched the CP group in terms of demographic characteristics. Finally, we conducted meta-regressions to assess the effects of continuous variables such as the sex ratio of the sample and the year of publication.

## 3. Results

### 3.1. Search Results

**Figure 1:**
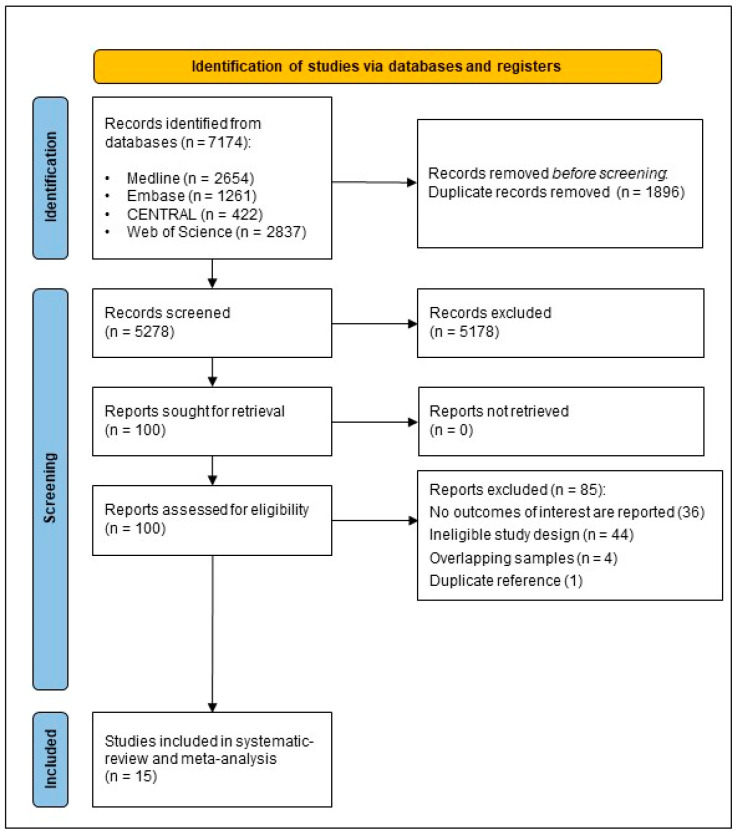
PRISMA flow diagram.

A total of 7174 records were identified in the databases. After the removal of 1896 duplicates, 5278 titles and abstracts were screened, of which 5178 were excluded for not meeting the eligibility criteria. Out of the remaining 100 studies, 85 were excluded based on the full text, resulting in 15 studies that were eligible for inclusion (Figure 1). We found strong agreement between reviewers in selecting studies based on full texts (Cohen’s kappa = 0.92). The characteristics of the included studies are shown in Table 1 [25,26,27,28,29,30,31,32,33,34,35,36,37,38,39].

### 3.2. Characteristics of the Studies Included

The studies were published between 2003 and 2023. Six studies reported data from Europe, five from North America, two from South America, and one from Asia and one from Australia. The number of cerebral paretic individuals examined in the 15 articles ranged from 8 to 76, with a total of 470 participants. Thirteen studies reported data on children, one on adults, and one investigated a mixed sample. The mean age of the cerebral paretic participants ranged from 4.9 to 34.2.

The number of typically developing participants in the control group was 536 in total, with sample sizes ranging between 8 and 89 participants in the studies. The age of the control group was similar to that of the CP group, with the mean age ranging from 4.9 to 34.7.

### 3.3. Results of Meta-Analysis

#### 3.3.1. Overall Differences

**Figure 2:**
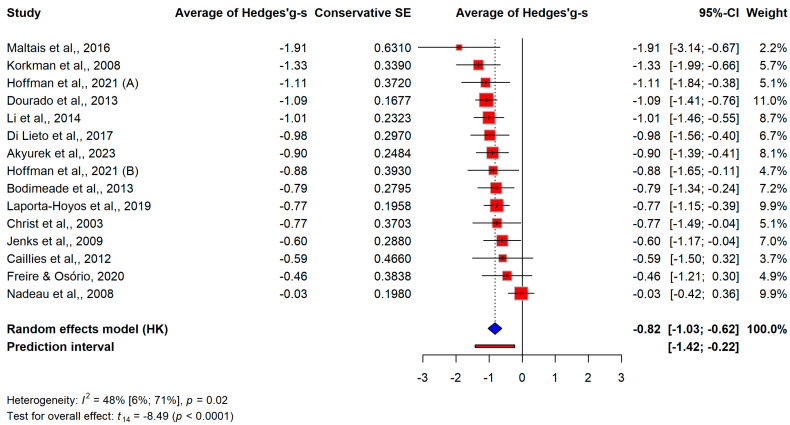
Comparison of overall executive functions between CP and control groups [25,26,27,28,29,30,31,32,33,34,35,36,37,38,39].

There was a large difference in CP patients’ executive function skills over the 15 studies that we could include (g+ = −0.82, 95% CI [−1.03; −0.62]). There was modest heterogeneity among the studies (I^2^ = 48%, 95% CI [6%; 71%], *p* = 0.02) as shown in Figure 2.

#### 3.3.2. Differences on the Components

**Figure 3:**
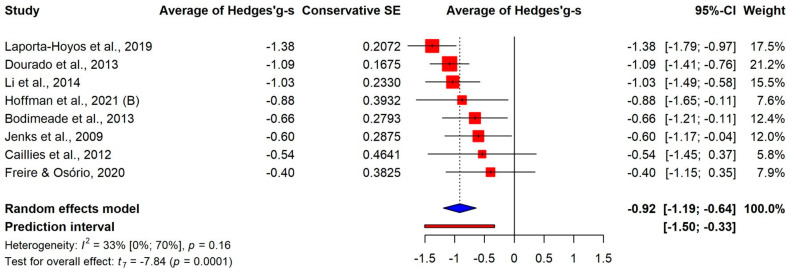
Comparison of working memory between CP and control groups [26,27,30,31,33,34,36,37].

**Figure 4:**
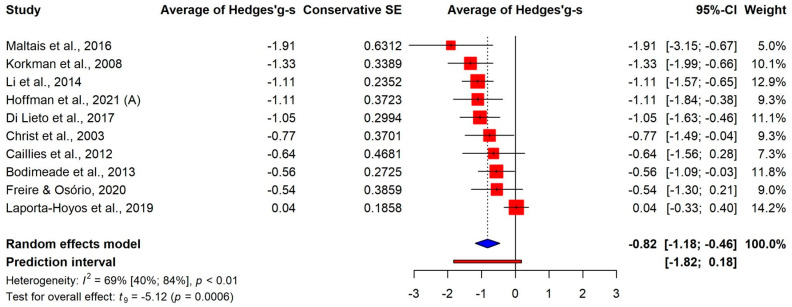
Comparison of inhibitory control between CP and control groups [26,27,28,29,31,32,35,36,37,38].

**Figure 5:**
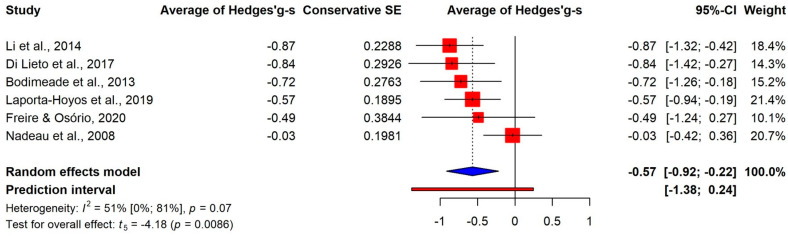
Comparison of cognitive flexibility between CP and control groups [26,29,31,36,37,39].

We examined the results for different components of executive function skills as well. There were large average differences in working memory (k = 8, g+ = −0.92, 95% CI [−1.19; −0.64]) and inhibitory control (k = 10, g+ = −0.82, 95% CI [−1.18; −0.46]), and a medium-sized effect was found for cognitive flexibility (k = 6, g+ = −0.57, 95% CI [−0.92; −0.22]), as shown in Figure 3, Figure 4 and Figure 5. It should be noted that the confidence intervals of the three estimates showed a large overlap. Additionally, a multivariate meta-analysis using a robust covariance estimate provided similar estimates for all the three components with similar standard errors.

We found large differences for both visuospatial working memory (k = 3, g+ = −1.20, 95% CI [−1.89; −0.52]) and verbal working memory (k = 6, g+ = −0.83, 95% CI [−1.21; −0.46]) (see Figure S1). When inspecting differences in the components of cognitive flexibility, we found a medium-sized difference for shifting tasks (k = 6, g+ = −0.53, 95% CI [−0.87; −0.19]), whereas the two studies using the planning task showed a medium-to-large difference (g+ = −0.71, 95% CI [−3.05; 1.62]) (see Figure S2).

#### 3.3.3. Permanent Deficit or Developmental Delay

To investigate the research question of whether a deficit in EF skills is a developmental delay, and thus whether the difference compared to a control group decreases as participants age or whether it is a persistent deficit, we examined the mean age of participants in the primary studies in a meta-regression. Additionally, we categorized the primary studies by the age range of the participants: children (early childhood (4–6.99 years old), middle childhood (7–13 years old) and adolescence (14–18 years old)) and adults, to pool effect sizes for the different age groups. Some studies applied a wider age range and could not be categorized.

The mean age of the participants did not have a significant effect on the effect sizes (coefficient: 0.0007, *p* = 0.9667). Furthermore, we found significant, large differences for both samples of children (k = 13, g+ = −0.83, 95% CI [−1.08; −0.58]) and the one sample of adults (k = 1, g+ = −0.88, 95% CI [−1.65; −0.11]). The one study that could not be categorized showed a significant medium-to-large difference (k = 1, g+ = −0.77, 95% CI [−1.15; −0.39]) (see Figure S3). Component differences can be found in Figures S4–S6 in the Supplementary Materials.

More specifically, there was a large, although not significant, difference in the two studies focusing on early childhood (k = 2, g+ = −0.91, 95% CI [−6.43; 4.61]). In contrast, we found a significant, medium-sized difference in middle childhood (k = 5, g+ = −0.73, 95% CI [−1.30; 0.17]). There was a large, but non-significant difference in the one study that examined adolescents (g+ = −1.11, 95% CI [−1.84; −0.38]). Studies that could not be categorized within the child category showed a large, significant difference (k = 5, g+ = −0.85, 95% CI [−1.25; −0.45]) (see Figure S7). Component differences can be found in Figures S8–S10 in the Supplementary Materials.

#### 3.3.4. Further Moderator Analyses

There was a significantly large difference for the spastic CP groups (k = 14, g+ = −0.83, 95% CI [−1.06; −0.60]) and a significantly large difference for a dyskinetic CP group (k = 1, g+ = −0.77, 95% CI [−1.15; −0.39]) (see Figure S11). The effect of diagnosis on the individual components of executive functions was investigated separately (see Figures S12–S14).

We found a non-significant, large difference in studies focusing on individuals with diplegia (k = 3, g+ = −0.95, 95% CI [−1.98; 0.07]), whereas a medium-sized difference was found in studies focusing on mixed spastic groups (k = 3, g+ = −0.51, 95% CI [−1.91; 0.88]) (see Figure S15).

We could not test the variation in the severity of CP between primary studies as a moderator. Notably, even for mild cases (where at least 80% of the participants were in GMFCS levels 1–3), a mean difference of −0.80 (k = 7, 95% CI [−1.28; −0.32]) was found. No article was published for GMFCS in which only participants with severe movement stage levels took part. Finally, the one study that included participants at all different levels of the GMFCS showed a large difference, too (g+ = −0.77, 95% CI [−1.15; −0.39]) (see Figure S16). The effect of GFMCS on the individual components of executive functions was investigated separately (see Figures S17–S19).

Similarly, we found similar-sized differences regardless of MACS level severity (see Figure S20). Articles in which at least 80% of participants with CP were classified as mild cases (MACS level 1 or 2) showed a large difference (k = 4, g+ = −0.85, 95% CI [−1.24; −0.45]). The two articles that included participants at different levels of the MACS also showed large differences (g+ = −0.90, 95% CI [−1.39; −0.41]) and (g+ = −0.77, 95% CI [−1.15; −0.39]). No article was identified in which at least 80% of participants were in the severe category (MACS level III, IV, V). For a comparison of EF components using MACS results, see Figures S21–S23.

We also examined the results in terms of whether the primary studies reported whether the control group and the CP group matched on variables such as age and sex (see Figure S24). There was a large difference (ꭕ^2^(1) = 7.59, *p* = 0.006) in studies that did not report the matching of the control group and the CP group (k = 6, g+ = −1.07, 95% CI [−1.32; −0.83]), whereas only a medium-sized difference was found in the studies that reported matching (k = 9, g+ = −0.67, 95% CI [−0.93; −0.41]). More specifically, large differences were found in working memory in both the one study that reported no matching (k = 1, g+ = −1.09, 95% CI [−1.41; −0.76]) and those that did report matching (k = 7, g+ = −0.86, 95% CI [−1.19; −0.53]) (see Figure S25). For inhibitory control, there was a large difference (ꭕ^2^(1) = 4.06, *p* = 0.04) between studies reporting no matching (k = 4, g+ = −1.16, 95% CI [−1.77; −0.56]), whereas there was a medium-sized effect between studies that did report matching (k = 6, g+ = −0.63, 95% CI [−1.10; −0.15]) (see Figure S26). Considering flexibility, we only found studies that reported matching. These studies showed a figure with a medium-sized average difference (k = 6, g+ = −0.57, 95% CI [−0.92; −0.22]) (see Figure S27).

Results for further moderators such as sex, year of publication and place of data collection can be found in the Supplementary Materials (see Table S1, Figures S28 and S29).

### 3.4. Publication Bias and Quality Assessment

There was no publication bias identified based on the visual inspection of the funnel plots and the Egger test performed (*p* = 0.44) (See Figures S30–S33).

**Figure 6:**
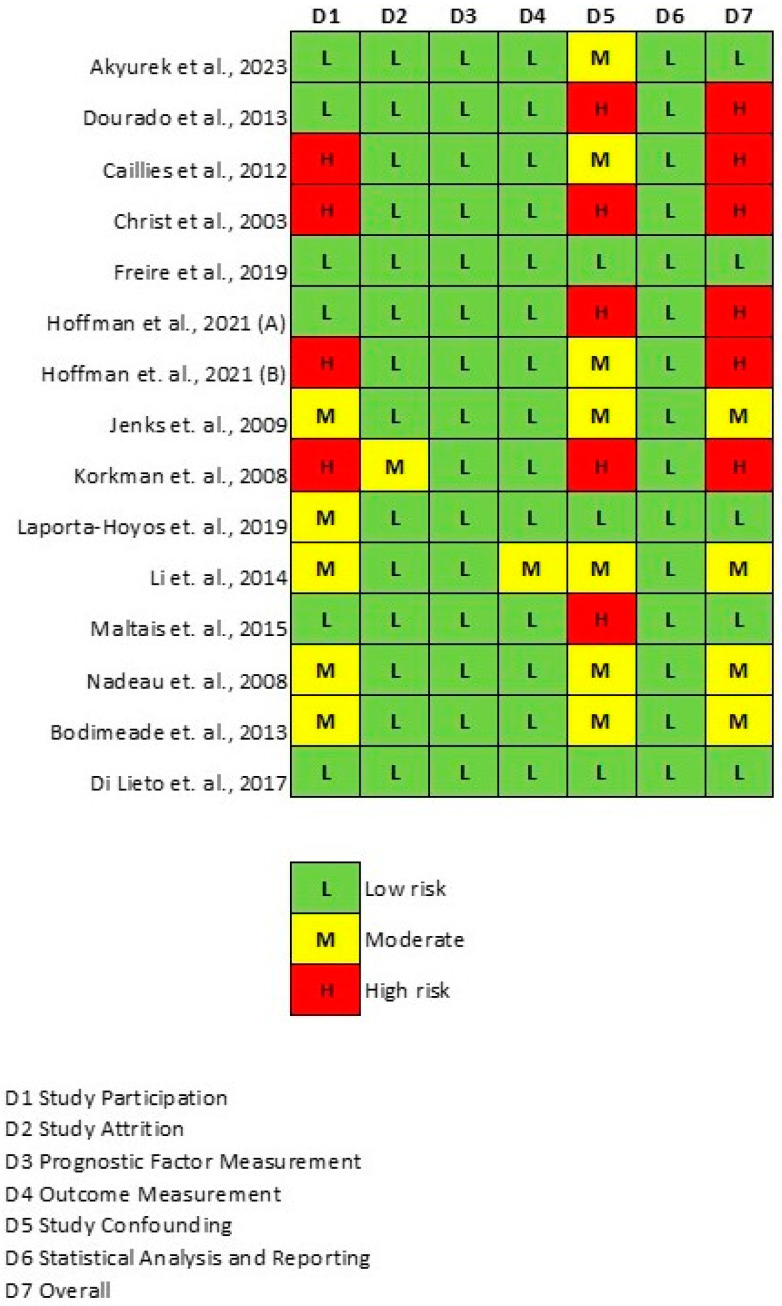
Result of the risk of bias assessment [25,26,27,28,29,30,31,32,33,34,35,36,37,38,39].

The 15 articles [25,26,27,28,29,30,31,32,33,34,35,36,37,38,39] include 6 high-, 4 moderate- and 5 low-risk studies. For further details see Figure 6.

## 4. Discussion

In this study, we confirmed in line with the literature [13,14,15,16] that individuals with CP experienced a significant deficit in EF skills, and, in fact, we found that there was an average difference of almost a standard deviation between individuals with CP and typically developing participants. Similarly large deficits were found in working memory and inhibitory control skills, whereas a medium-sized difference was found in cognitive flexibility. It should be noted that the confidence intervals were large and overlapping, so we cannot conclude that individuals with CP experience a smaller deficit in cognitive flexibility than in working memory and inhibitory control. Furthermore, a methodological issue was found to have an effect: studies that matched [26,27,29,31,33,34,36,37,39] the control group to the CP group by age and sex found a smaller difference than studies that did not report on such matching [25,28,30,32,35,38]. Thus, it is also important to note that all studies reporting on a measure of cognitive flexibility applied to the matching of controls and the CP group, whereas studies on working memory and inhibitory control were mixed in this regard. This might also explain why only medium-sized differences were found in the cognitive flexibility between individuals with CP and controls. In fact, when we pooled the studies that applied matching, only a medium-sized effect was found for inhibitory control; however, the large effect on working memory remained. Additionally, similar effects were found for verbal [26,27,31,34,36] and visuospatial working memory [26,27,28,29,31,32,35,36,37,38,39], and for measures of shifting [26,29,31,36,37,39] and planning [26,36]. In sum, we confirmed the results of previous studies [13,14,15,16] showing substantial deficits in EF skills in CP. Moreover, we found that all aspects of EF skills were affected. 

We were particularly interested in the effects of age on the effect size in order to investigate whether the deficit decreased or remained stable over development [13]. The mean of the sample was not a significant predictor of the difference between the two groups. Considering the limitations of testing in the mean age of the sample, which disregarded the age range, we examined the results in different age groups as well. A large effect size was found between studies focusing on early childhood samples, whereas middle childhood samples showed only medium-sized effects; however, large effects were found in the one study on adolescents and the one study with an adult sample. These results are based on a couple of studies and are thus purely preliminary; however, they might suggest that the deficit in EF skills in CP is permanent. Future meta-analyses should revisit the question when more studies are available.

Similar effects were found when different types of diagnosis were taken into account. Interestingly, the effects do not seem to be affected by the severity of the diagnosis: large differences are found even in samples of mild cases. These highlight the universality of EF deficits in CP. 

### 4.1. Strengths and Limitations

The availability of sufficient articles to answer the research questions allowed us to carry out the first meta-analysis on this topic, which did not only look at overall executive functions, but also at the different components, while preliminary results on potential moderators could also be inspected. We also performed subgroup analyses to reduce heterogeneity.

The current meta-analysis has several limitations worth highlighting. Only 15 studies were available; thus, not all moderator analyses could be adequately conducted. Also, we pooled data from all age groups. This approach has its limitations, as different tests of EF skills were used for different age groups, and it was not clear how comparable they were. Finally, as aggregate data were used to analyze age subgroups, the results should be interpreted with caution due to ecological bias.

### 4.2. Implication for Practice 

The results reinforce the notion that the CP population needs developmental programs to improve their executive functions.

### 4.3. Implication for Research

Future studies should focus on how various developmental programs could narrow the gap between the typically developing and CP population, and further research would give us a better understanding of the executive functions of adult CP. Finally, on the basis of the results, it is highly recommended for future research to carry out thorough matching of the control group on a range of variables.

## 5. Conclusions

The present meta-analysis highlighted that all components of EF skills were substantially affected in CP. We also presented quantifiable results showing the universality of this deficit regardless of diagnosis type or severity of CP and that these do not decrease over time. According to the available data, our results indicated a strong need for intervention to improve executive functions in CP patients.

## Figures and Tables

**Table 1:** Characteristics of included studies in the meta-analysis and systematic review.

Author, Publication Year	Country	Age M (Year)	Type of CP	No. of CP’s Patients	GMFCS *	MACS **	Outcome Measure
Akyurek et. al., 2023 [25]	Turkey	8.5	Spastic	22	mild	mix	Executive Function (mix): 1. Executive Function and Occupational Routines Scale (EFFORTS)
Bodimeade et al., 2013 [26]	Australia	11.09	Spastic	46	mild	mild	Working memory (4 measures): 1. Digit span (backward) 2. Rey/Osterrieth ComplexFigure task 3. Code transmission test 4. Verbal fluency Inhibitory control (1 measure): 1. Stroop Cognitive flexibility (2 measures): 1. Tower of London 2. Stroop Executive functions (mix): 1. Behavior Rating Inventory of Executive Function (BRIEF)
Caillies et al., 2012 [27]	France	9.3	Spastic	10	no data	no data	Working memory (2 measures): 1. Digit span (backward) 2. Letter–Number Sequencing Inhibitory control (2 measures): 1. Stroop 2. Knock–Tap
Christ et al., 2003 [28]	USA	13.9	Spastic	13	no data	no data	Inhibitory control (3 measures): 1. Stroop 2. Stimulus–response reversal task 3. Antisaccade task
Di Lieto et al., 2017 [29]	Italy	8.58	Spastic	19	mix	mild	Inhibitory control (1 measure): 1. NEPSY Statue and Auditory Attention and Response Set subtest, part A Cognitive flexibility (1 measure): 1. NEPSY Statue and Auditory Attention and Response Set subtest, part A
Dourado et.al., 2013 [30]	Brazil	8.9	Spastic	76	mild	no data	Working memory (3 measures): 1. Corsi Blocktapping Test (backward) 2. Digit span (backward) 3. Rey/Osterrieth ComplexFigure task
Freire et al., 2019 [31]	Brazil	4.97	Spastic	14	mild	mild	Working memory (2 measures): 1. Reverse words Inhibitory control (1 measure): 1. Stroop Cognitive flexibility (1 measure): 1. Trail-making Test
Hoffman et al. (A), 2021 [32]	USA	15.7	Spastic	14	mild	mix	Inhibitory control (1 measure): 1. Eriksen Flanker task
Hoffman et al. (B), 2021 [33]	USA	34.2	Spastic	13	mix	no data	Working memory (2 measure): 1. Sternberg-type working memory task
Jenks et al., 2009 [34]	Netherlands	7.0	Spastic	57	no data	no data	Working memory (1 measure): 1. Digit span (backward)
Korkman et al., 2008 [35]	Finland	5.82	Spastic	12	no data	no data	Inhibitory control (1 measure): 1. NEPSY Statue and Auditory Attention and Response Set subtest, part A
Laporta-Hoyos et al., 2019 [36]	Spain	20.5	Spastic	52	mix	mix	Working memory (2 measures): 1. Corsi Blocktapping Test (backward) 2. Digit span (backward) Inhibitory control (1 measure): 1. Stop Signal task Cognitive flexibility (2 measures): 1. Wisconsin Card Sorting Test (WCST) 2. Stockings of Cambridge (SOC) test from the CANTAB.
		20.5	Dyskinetic	20	mix	mix	
Li et al., 2014 [37]	China	10.40	Spastic	42	no data	no data	Working memory (1 measure): 1. Running Memory Inhibitory control (1 measure): 1. Day-night task Cognitive flexibility (1 measure): 1. Plus–minus task
Maltais et al., 2015 [38]	Canada	11.4	Spastic	8	mild	no data	Inhibitory control (1 measure): 1. Stroop
Nadeau et al., 2008 [39]	Canada	11.4	Spastic	52	mild	no data	Cognitive flexibility (1 measure): 1. Wisconsin Card Sorting Test (WCST)

* GMFCS: Mild = at least 80% of the CP participants belong to GMFCS level I, II, III according to the article. Severe = at least 80% of the CP participants belong to GMFCS level IV, V according to the article. Mix = CP participants were at levels GMFCS I–V according to the article. ** MACS: Mild = at least 80% of the participants belong to MACS level I, II according to the article. Severe = at least 80% of the CP participants belong to MACS level III, IV, V according to the article. Mix = CP participants were at levels MACS I-V according to the article.

## Data Availability

The data presented in this study are available in this article and Supplementary Materials.

## Supplementary Materials

The following supporting information can be downloaded at: https://www.mdpi.com/article/10.3390/jcm13071867/s1. Supplemental S1: Operational definitions. Supplemental S2: Data sources and search strategy. Supplemental S3: Quality Assessment. Supplemental S4: Differences on the components. Supplemental S5: Permanent deficit or developmental delay. Supplemental S6: Type and severity of CP. Supplemental S7: Methodological differences. Supplemental S8: Publication Bias. Table S1: Results of meta-regression analyses for methodological differences. Figure S1: Comparison of visuospatial and verbal working memory between CP and control groups. Figure S2: Comparison of planning and shifting abilities between CP and control groups. Figure S3: Comparison of effect sizes by adult and child samples. Figure S4: Comparison of effect sizes on working memory by adult and child samples. Figure S5: Comparison of effect sizes on inhibitory control by adult and child samples. Figure S6: Comparison of effect sizes on flexibility by adult and child samples. Figure S7: Comparison of effect sizes by age groups of children. Figure S8: Comparison of effect sizes on working memory by age groups of children. Figure S9: Comparison of effect sizes on inhibitory control by age groups of children. Figure S10: Comparison of effect sizes on flexibility by age groups of children. Figure S11: Comparison of effect sizes by diagnosis. Figure S12: Comparison of effect sizes on working memory by diagnosis. Figure S13: Comparison of effect sizes on inhibitory control by diagnosis. Figure S14: Comparison of effect sizes on flexibility by diagnosis. Figure S15: Comparison of effect sizes according to diagnosis. Figure S16: Comparison of effect sizes by GMFCS. Figure S17: Comparison of effect sizes on working memory by GMFCS. Figure S18: Comparison of effect sizes on inhibitory control by GMFCS. Figure S19: Comparison of effect sizes on flexibility by GMFCS. Figure S20: Comparison of effect sizes by MACS. Figure S21: Comparison of effect sizes on working memory by MACS. Figure S22: Comparison of effect sizes on inhibitory control by MACS. Figure S23: Comparison of effect sizes on flexibility by MACS. Figure S24: Comparison of effect sizes by matching. Figure S25: Comparison of effect sizes on working memory by matching of CP and control groups. Figure S26: Comparison of effect sizes on inhibitory control by matching of CP and control groups. Figure S27: Comparison of effect sizes on flexibility by matching of CP and control groups. Figure S28: Comparison of effect sizes by continent. Figure S29: Comparison of effect sizes between North-America and Europe. Table S1: Results of meta-regression analyses for methodological differences. Figure S30: Funnel plot of studies reporting on measure of executive functions. Figure S31: Funnel plot of studies reporting on a measure of working memory. Figure S32: Funnel plot of studies reporting on a measure of inhibitory control. Figure S33: Funnel plot of studies reporting on a measure of flexibility.

## References

[1] Sellier E., Platt M.J., Andersen G.L., Krägeloh-Mann I., De La Cruz J., Cans C., Surveillance of Cerebral Palsy Network (2016). Decreasing prevalence in cerebral palsy: A multi-site European population-based study, 1980 to 2003. Dev. Med. Child. Neurol..

[2] Van Naarden Braun K., Doernberg N., Schieve L., Christensen D., Goodman A., Yeargin-Allsopp M. (2016). Birth Prevalence of Cerebral Palsy: A Population-Based Study. Pediatrics.

[3] Rosenbaum P., Paneth N., Leviton A., Goldstein M., Bax M., Damiano D., Dan B., Jacobsson B. (2007). A report: The definition and classification of cerebral palsy April 2006. Dev. Med. Child Neurol..

[4] Piscitelli D., Ferrarello F., Ugolini A., Verola S., Pellicciari L. (2021). Measurement properties of the Gross Motor Function Classification System, Gross Motor Function Classification System-Expanded & Revised, Manual Ability Classification System, and Communication Function Classification System in cerebral palsy: A systematic review with meta-analysis. Dev. Med. Child. Neurol..

[5] Diamond A. (2013). Executive functions. Annu. Rev. Psychol..

[6] Allan N.P., Hume L.E., Allan D.M., Farrington A.L., Lonigan C.J. (2014). Relations between inhibitory control and the development of academic skills in preschool and kindergarten: A meta-analysis. Dev. Psychol..

[7] Jacob R., Parkinson J. (2015). The potential for school-based interventions that target executive function to improve academic achievement: A review. Rev. Educ. Res..

[8] Spiegel J.A., Goodrich J.M., Morris B.M., Osborne C.M., Lonigan C.J. (2021). Relations between executive functions and academic outcomes in elementary school children: A meta-analysis. Psychol. Bull..

[9] Rhoades B.L., Greenberg M.T., Domitrovich C.E. (2009). The contribution of inhibitory control to preschoolers’ social–emotional competence. J. Appl. Dev. Psychol..

[10] Li Q., Liu P., Yan N., Feng T. (2020). Executive Function Training Improves Emotional Competence for Preschool Children: The Roles of Inhibition Control and Working Memory. Front. Psychol..

[11] Hughes C., Ensor R. (2008). Does executive function matter for preschoolers’ problem behaviors?. J. Abnorm. Child. Psychol..

[12] Miyake A., Friedman N.P., Emerson M.J., Witzki A.H., Howerter A., Wager T.D. (2000). The unity and diversity of executive functions and their contributions to complex “Frontal Lobe” tasks: A latent variable analysis. Cogn. Psychol..

[13] Bottcher L. (2010). Children with spastic cerebral palsy, their cognitive functioning, and social participation: A review. Child. Neuropsychol..

[14] Pereira A., Lopes S., Magalhães P., Sampaio A., Chaleta E., Rosário P. (2018). How Executive Functions Are Evaluated in Children and Adolescents with Cerebral Palsy? A Systematic Review. Front. Psychol..

[15] Straub K., Obrzut J.E. (2009). Effects of cerebral palsy on neuropsychological function. J. Dev. Phys. Disabil..

[16] Weierink L., Vermeulen R.J., Boyd R.N. (2013). Brain structure and executive functions in children with cerebral palsy: A systematic review. Res. Dev. Disabil..

[17] Best J.R., Miller P.H. (2010). A developmental perspective on executive function. Child. Dev..

[18] Moher D., Liberati A., Tetzlaff J., Altman D.G., PRISMA Group (2009). Preferred reporting items for systematic reviews and meta-analyses: The PRISMA statement. PLoS Med..

[19] Alloway T.P., Alloway R.G. (2010). Investigating the predictive roles of working memory and IQ in academic attainment. J. Exp. Child. Psychol..

[20] Hayden J.A., van der Windt D.A., Cartwright J.L., Côté P., Bombardier C. (2013). Assessing bias in studies of prognostic factors. Ann. Intern. Med..

[21] Harrer M., Cuijpers P., Furukawa T.A., Ebert D.D. (2021). Doing Meta-Analysis with R: A Hands-On Guide.

[22] Higgins J.P.T., Thomas J., Chandler J., Cumpston M., Li T., Page M.J., Welch V.A. (2022). Cochrane Handbook for Systematic ReViews of Interventions Version 6.3 (updated February 2022). Cochrane. www.training.cochrane.org/handbook.

[23] Pustejovsky J.E., Tipton E. (2022). Meta-analysis with Robust Variance Estimation: Expanding the Range of Working Models. Prev. Sci..

[24] Gleser L.J., Olkin I., Cooper H., Hedges L.V., Valentine J.C. (2009). Stochastically dependent effect sizes. The Handbook of Research Synthesis and Meta-Analysis.

[25] Akyurek G., Gurbuz D., Irmak D. (2023). Comparison of the Executive Functions, Occupational Performance and Perceived Occupational Proficiency in Children with Neurodevelopmental Disorder. J. Occup. Ther. Sch..

[26] Bodimeade H.L., Whittingham K., Lloyd O., Boyd R.N. (2013). Executive function in children and adolescents with unilateral cerebral palsy. Dev. Med. Child. Neurol..

[27] Caillies S., Hody A., Calmus A. (2012). Theory of mind and irony comprehension in children with cerebral palsy. Res. Dev. Disabil..

[28] Christ S.E., White D.A., Brunstrom J.E., Abrams R.A. (2003). Inhibitory control following perinatal brain injury. Neuropsychology.

[29] Di Lieto M.C., Brovedani P., Pecini C., Chilosi A.M., Belmonti V., Fabbro F., Urgesi C., Fiori S., Guzzetta A., Perazza S. (2017). Spastic diplegia in preterm-born children: Executive function impairment and neuroanatomical correlates. Res. Dev. Disabil..

[30] Dourado M.R., Andrade P.M., Ramos-Jorge M.L., Moreira R.N., Oliveira-Ferreira F. (2013). Association between executive/attentional functions and caries in children with cerebral palsy. Res. Dev. Disabil..

[31] Freire T.C., Osório A. (2020). Executive functions and drawing in young children with cerebral palsy: Comparisons with typical development. Child. Neuropsychol..

[32] Hoffman R.M., Embury C.M., Lew B.J., Heinrichs-Graham E., Wilson T.W., Kurz M.J. (2021). Cortical oscillations that underlie visual selective attention are abnormal in adolescents with cerebral palsy. Sci. Rep..

[33] Hoffman R.M., Trevarrow M.P., Bergwell H.R., Embury C.M., Heinrichs-Graham E., Wilson T.W., Kurz M.J. (2021). Cortical oscillations that underlie working memory are altered in adults with cerebral palsy. Clin. Neurophysiol..

[34] Jenks K.M., de Moor J., van Lieshout E.C. (2009). Arithmetic difficulties in children with cerebral palsy are related to executive function and working memory. J. Child. Psychol. Psychiatry.

[35] Korkman M., Mikkola K., Ritari N., Tommiska V., Salokorpi T., Haataja L., Tammela O., Pääkkönen L., Olsén P., Fellman V. (2008). Neurocognitive test profiles of extremely low birth weight five-year-old children differ according to neuromotor status. Dev. Neuropsychol..

[36] Laporta-Hoyos O., Ballester-Plané J., Leiva D., Ribas T., Miralbell J., Torroja-Nualart C., Russi M.E., Toro-Tamargo E., Meléndez-Plumed M., Gimeno F. (2019). Executive function and general intellectual functioning in dyskinetic cerebral palsy: Comparison with spastic cerebral palsy and typically developing controls. Eur. J. Paediatr. Neurol..

[37] Li X., Wang K., Wu J., Hong Y., Zhao J., Feng X., Xu M., Wang M., Ndasauka Y., Zhang X. (2014). The link between impaired theory of mind and executive function in children with cerebral palsy. Res. Dev. Disabil..

[38] Maltais D.B., Gane C., Dufour S.K., Wyss D., Bouyer L.J., McFadyen B.J., Zabjek K., Andrysek J., Voisen J.I. (2016). Acute Physical Exercise Affects Cognitive Functioning in Children With Cerebral Palsy. Pediatr. Exerc. Sci..

[39] Nadeau L., Routhier M.E., Tessier R. (2008). The performance profile on the Wisconsin Card Sorting Test of a group of children with cerebral palsy aged between 9 and 12. Dev. Neurorehabil..

