# Supplementary material for: Comparison of Executive Function Skills between Patients with Cerebral Palsy and Typically Developing Populations: A Systematic Review and Meta-Analysis
Source: J Clin Med. 2024 Mar 24;13(7):1867. doi: 10.3390/jcm13071867 (PMC11012391; doi:10.3390/jcm13071867)
Supplement: Supplementary file 1 [file jcm-13-01867-s001.zip › jcm-2910500-supplementary.pdf]

SUPPLEMENTARY MATERIALS

Comparison of Executive Function Skills between Patients with Cerebral Palsy and Typically Developing Populations: A Systematic Review and Meta-Analysis

13March2024

Table of contents

No.	Title	Page no.
	**Materials and Methods**	
**1.**	Operational definitions	**1**
**2.**	Data sources and search strategy	**2**
**3.**	Quality Assessment	**2**
	**Results**	
**4.**	Differences on the components	**3**
**5.**	Permanent deficit or developmental delay	**4**
**6.**	Type and severity of CP	**8**
**7.**	Methodological differences	**15**
**8.**	Publication Bias	**19**

Materials and Methods

Supplemental S1 - Operational definitions

The Corsi Blocktapping Test [30,36], backward Digit span tasks [26,27,30,34,36], Rey/Osterrieth Complex Figure task [26,30], Letter–Number Sequencing [27], Reverse words [31], Sternberg-type working memory task [33], Running Memory [37], Verbal fluency [26], and the Code transmission test [26] were considered as measures of working memory. Tests that required short-term storage of information but no manipulation of that information such as forward span tests were considered to assess short-term memory and were not included.

Tests that required participants to inhibit either an automatized reaction or a previously learnt response were categorized as measures of inhibitory control: Stop signal task [36], Stroop [26-28,31,38], Antisaccade task [28], Day–night task [37], Knock–Tap [27], Stimulus–response reversal task [28], Eriksen Flanker task [32], NEPSY Statue and Auditory Attention and Response Set subtest, part A [29,35].

Tests of shifting abilities such as the Wisconsin Card Sorting Test [36,39], the Plus–minus task [37], and the Trail-making Test, part B [31] were coded as measures of cognitive flexibility. In

addition, tests of planning such as the Stockings of Cambridge test [36] and the Tower of London [26] were also categorized as cognitive flexibility.

Supplemental S2 - Data sources and search strategy

Search key: ("cognitive control" OR "behavi* control" OR "self-control" OR "effortful control" OR "self-regulat*" OR "executive functi*" OR "selective attention" OR "working memory" OR updating OR inhibit* OR planning OR shifting OR "delayed gratification") AND ("cerebral palsy" OR "cerebral paresis" OR "cerebral paretic").

Supplemental S3 - Quality Assessment

In the Study Participation domain (D1), the study was considered high risk if it did not report GMFCS or MACS classification. We considered the risk moderate, for example, if the study did not report the location and duration of recruitment. For the Study Attrition domain (D2), if more than 20% of the participants dropped out of the research but reported the underlying reason, it was considered moderate. The Prognostic Factor Measurement domain (D3) shows whether the test used to determine that a participant had CP is a valid method. For the Outcome Measurement domain (D4), if the method and setting of the outcome measure was not the same for all study participants, it was then coded to be a moderate risk. We considered the articles where no matching of the control group to the CP group was reported as high risk, and taken as moderate risk if the article did not report whether other neurological problems were excluded (see Study Confounding domain - D5). Statistical Analysis and Reporting domain (D6) was low risk if there is sufficient presentation of data to assess the adequacy of the analysis and there is no selective reporting of results.

Results

Supplemental S4 - Differences on the components

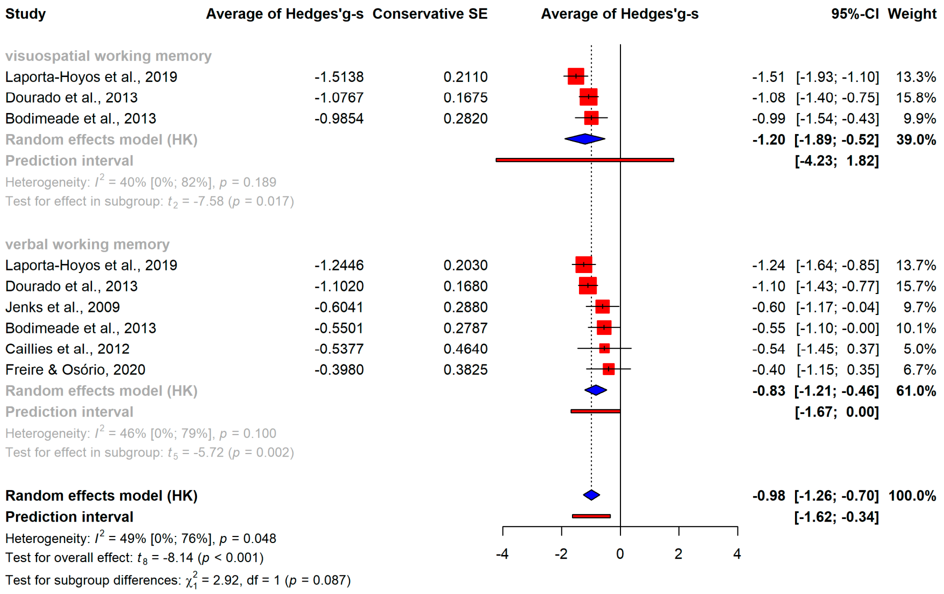

Figure S1. Comparison of visuospatial and verbal working memory between CP and control groups.

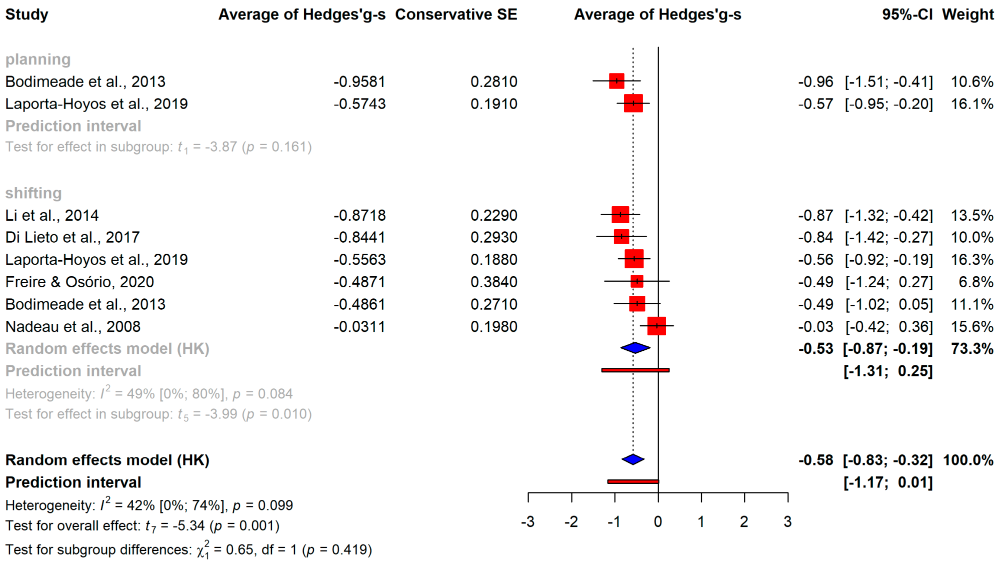

Figure S2. Comparison of planning and shifting abilities between CP and control groups.

Supplemental S5 - Permanent deficit or developmental delay

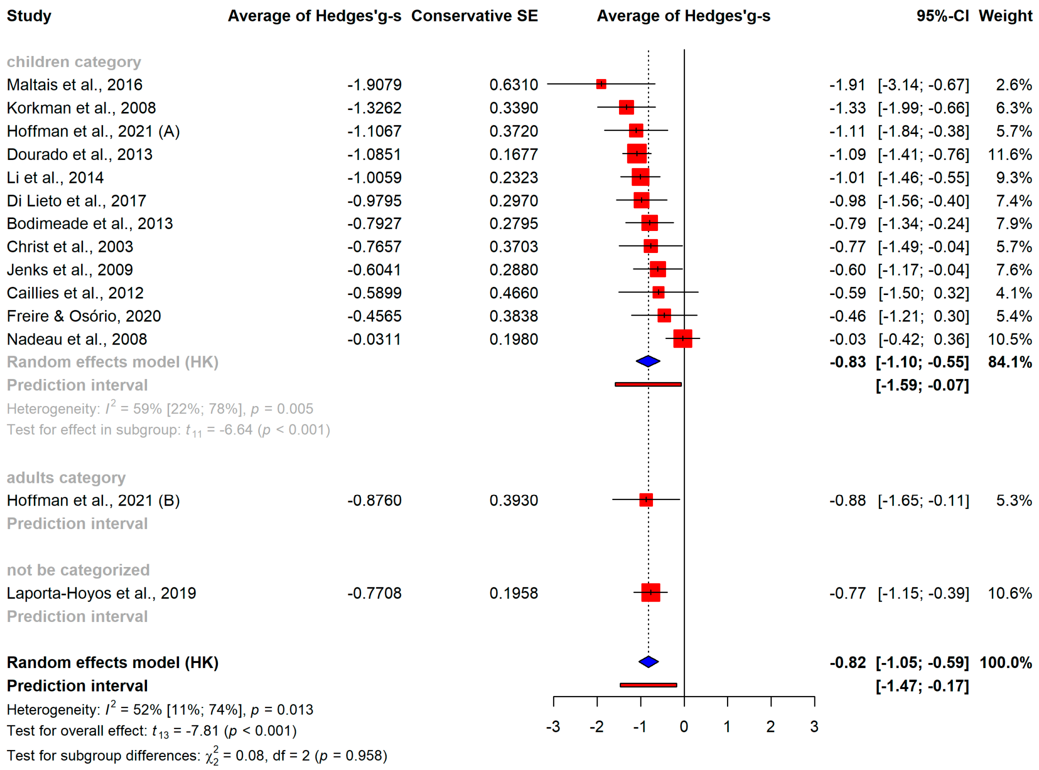

Figure S3. Comparison of effect sizes by adult and child samples.

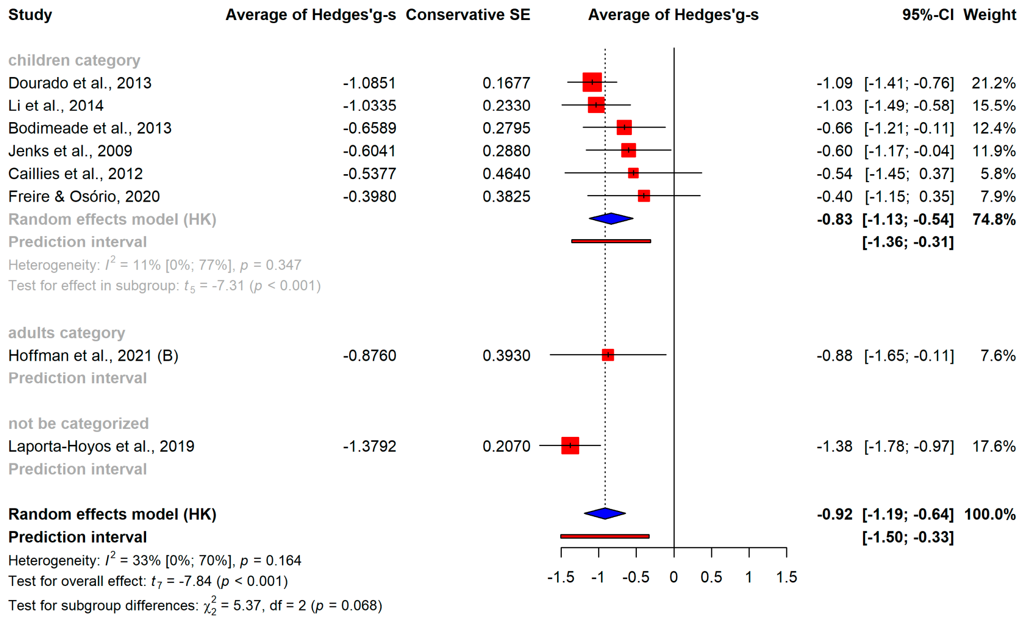

Figure S4. Comparison of effect sizes on working memory by adult and child samples.

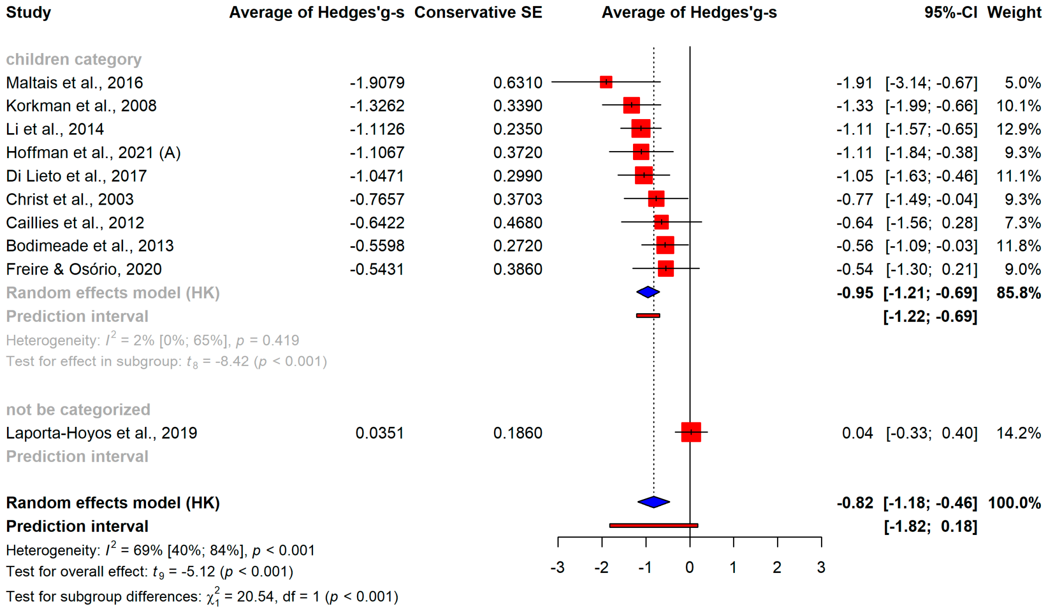

Figure S5. Comparison of effect sizes on inhibitory control by adult and child samples.

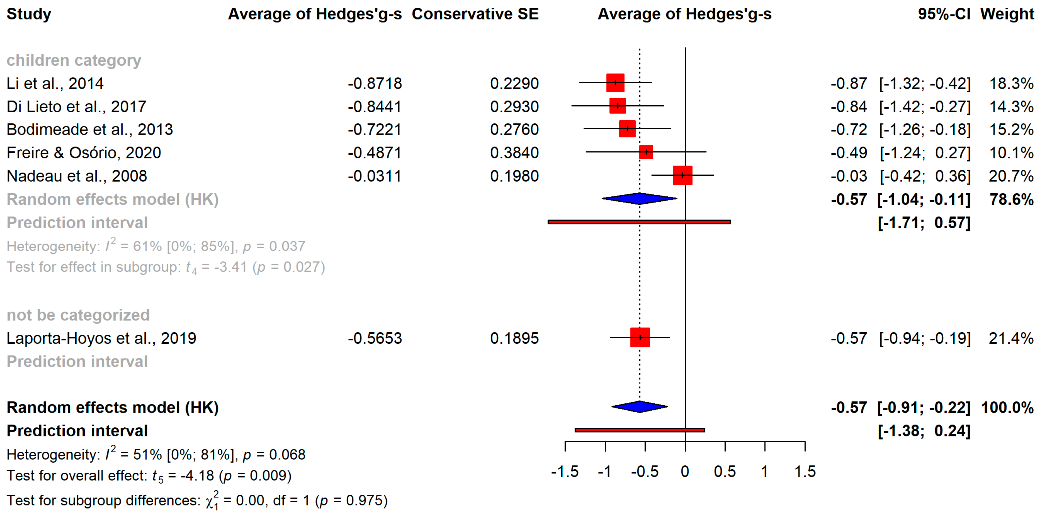

Figure S6. Comparison of effect sizes on flexibility by adult and child samples.

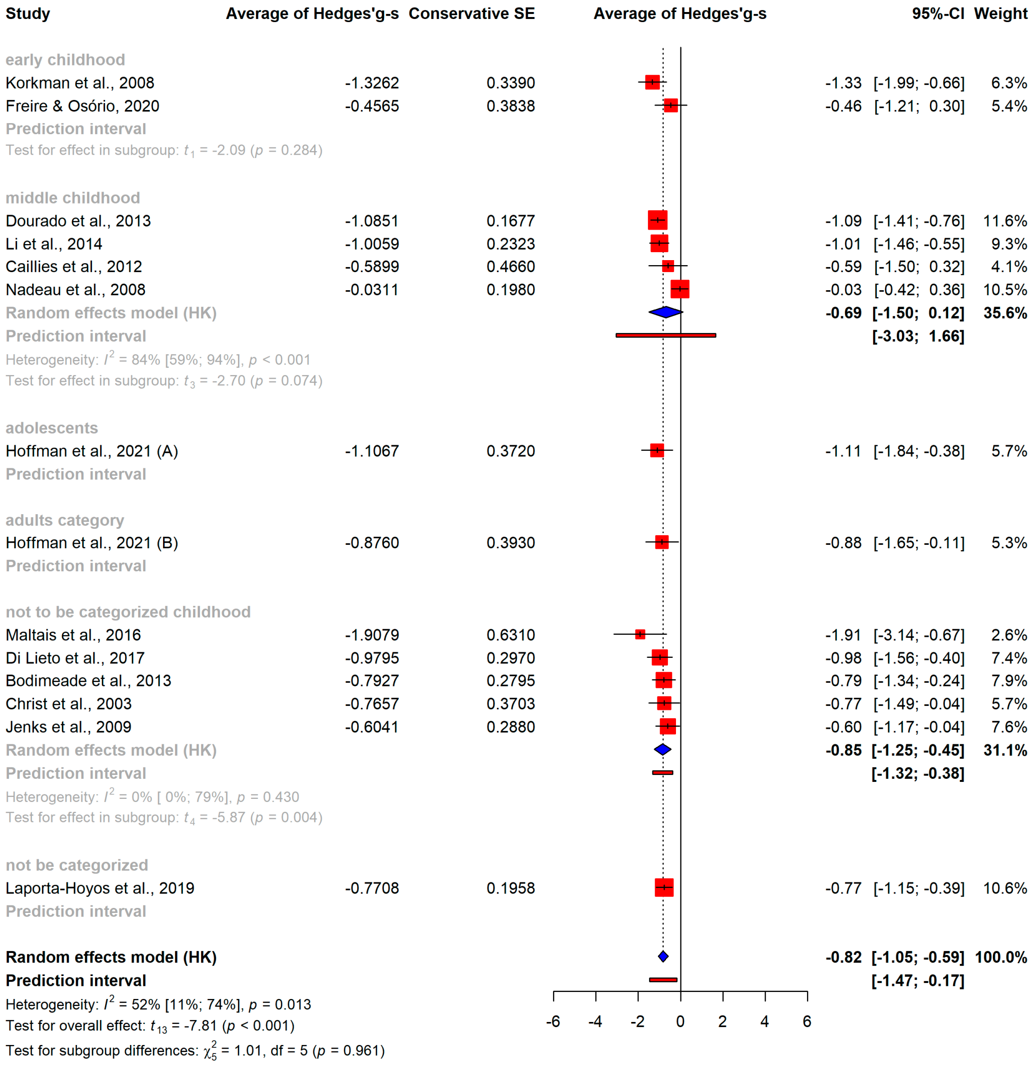

Figure S7. Comparison of effect sizes by age groups of children.

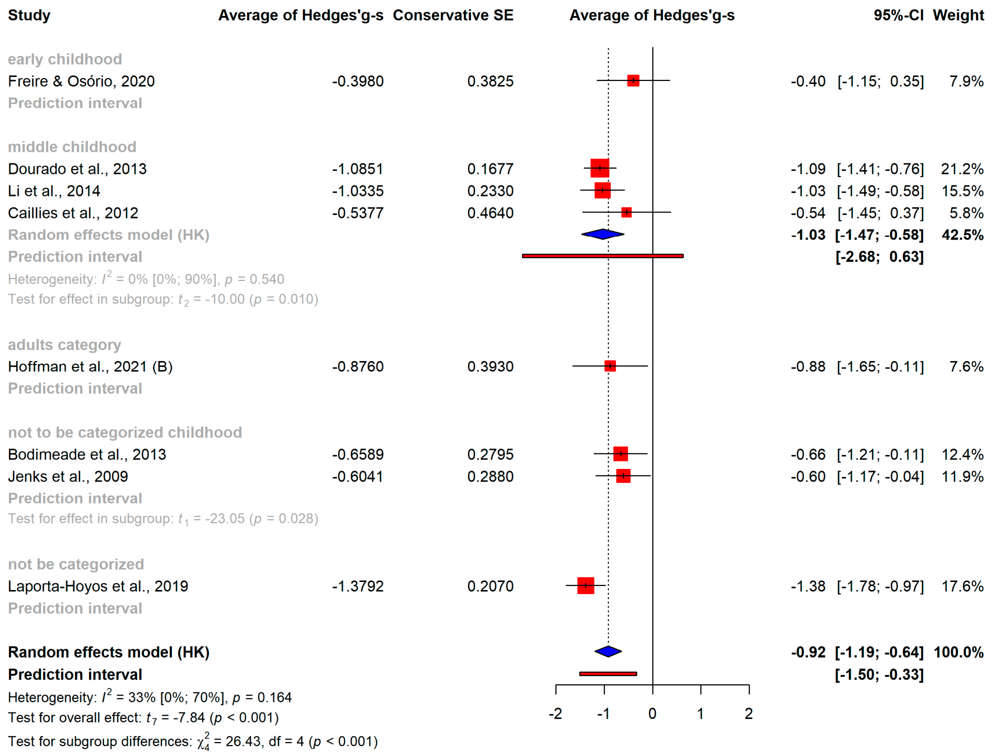

Figure S8. Comparison of effect sizes on working memory by age groups of children.

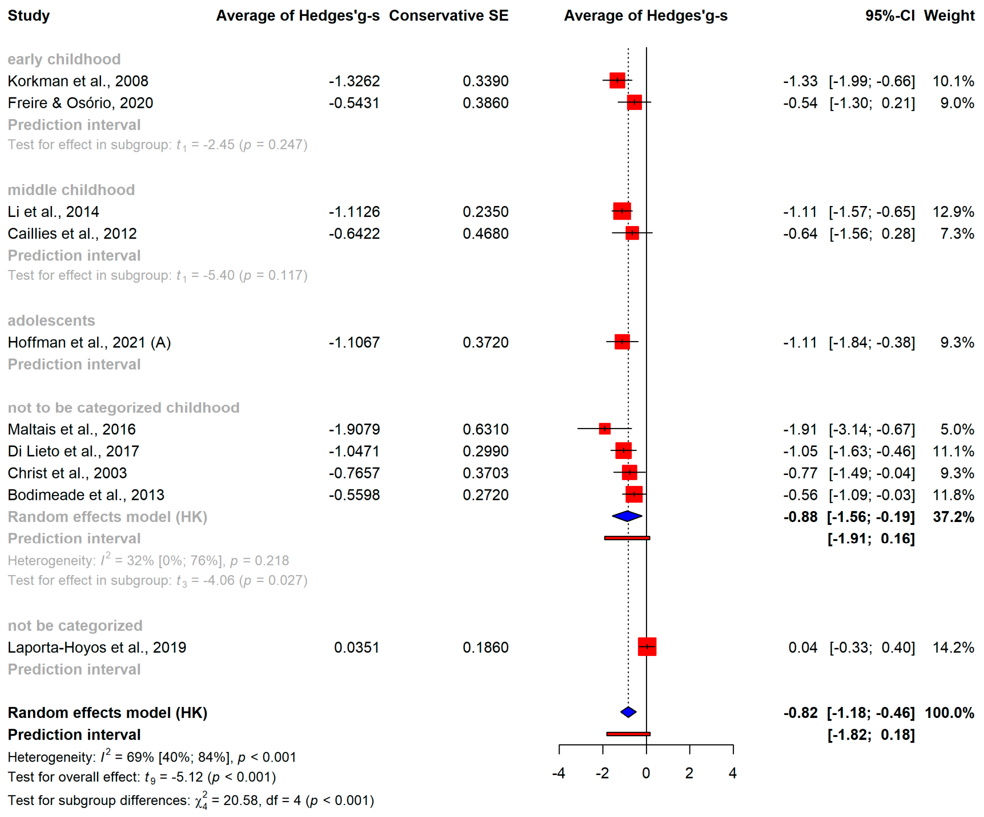

Figure S9. Comparison of effect sizes on inhibitory control by age groups of children.

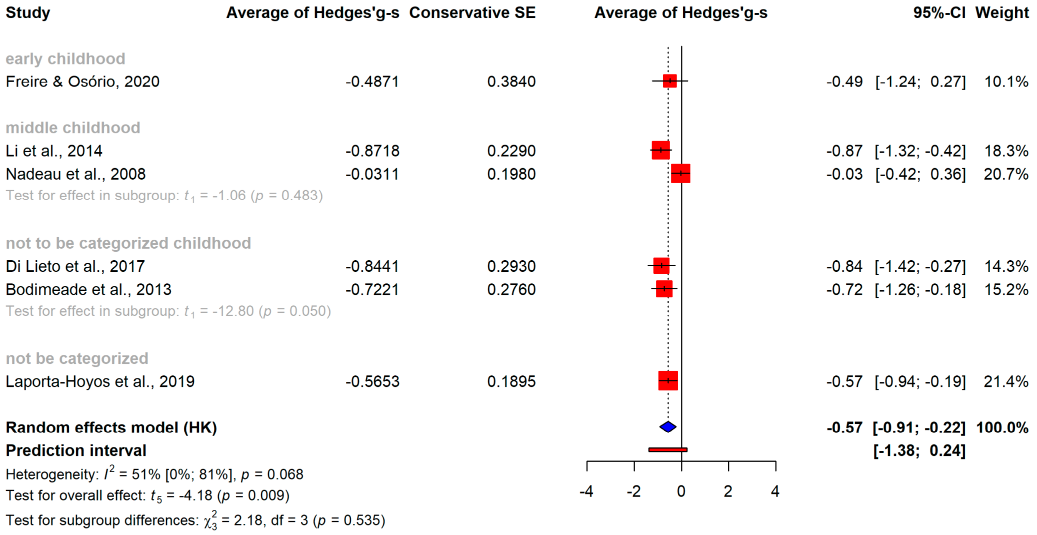

Figure S10. Comparison of effect sizes on flexibility by age groups of children.

Supplemental S6 - Type and severity of CP

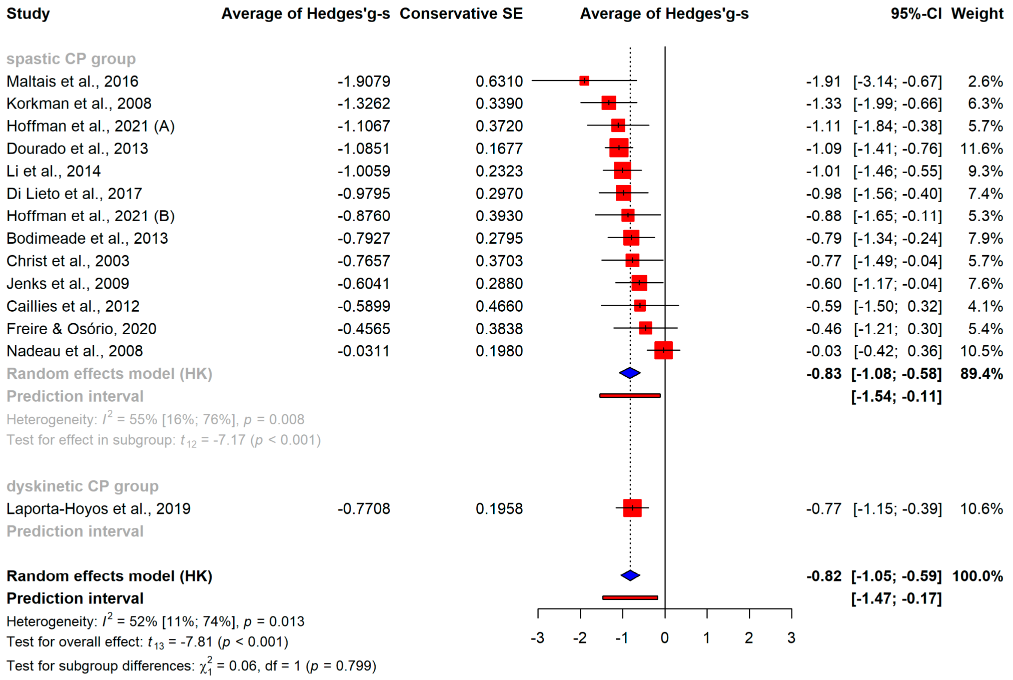

Figure S11. Comparison of effect sizes by diagnosis.

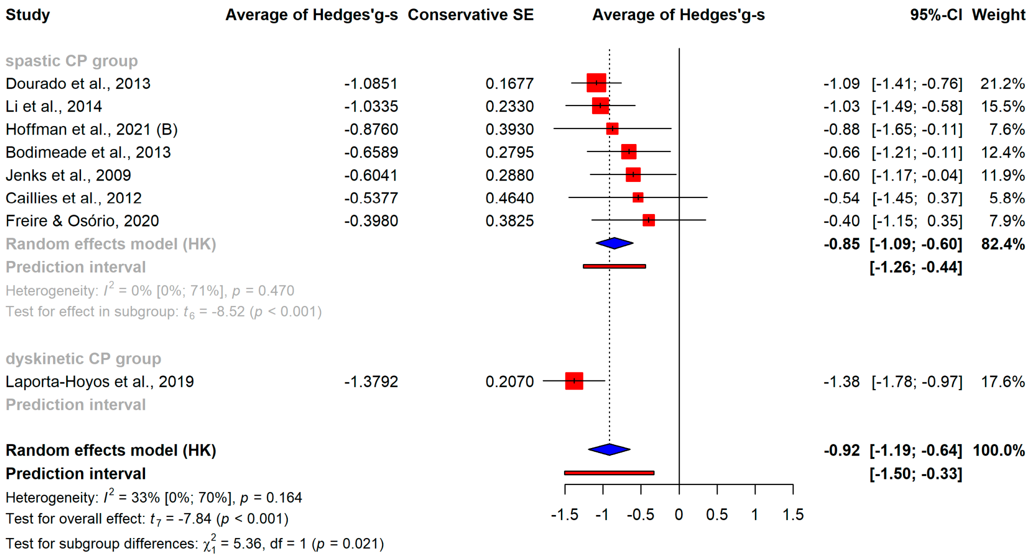

Figure S12. Comparison of effect sizes on working memory by diagnosis.

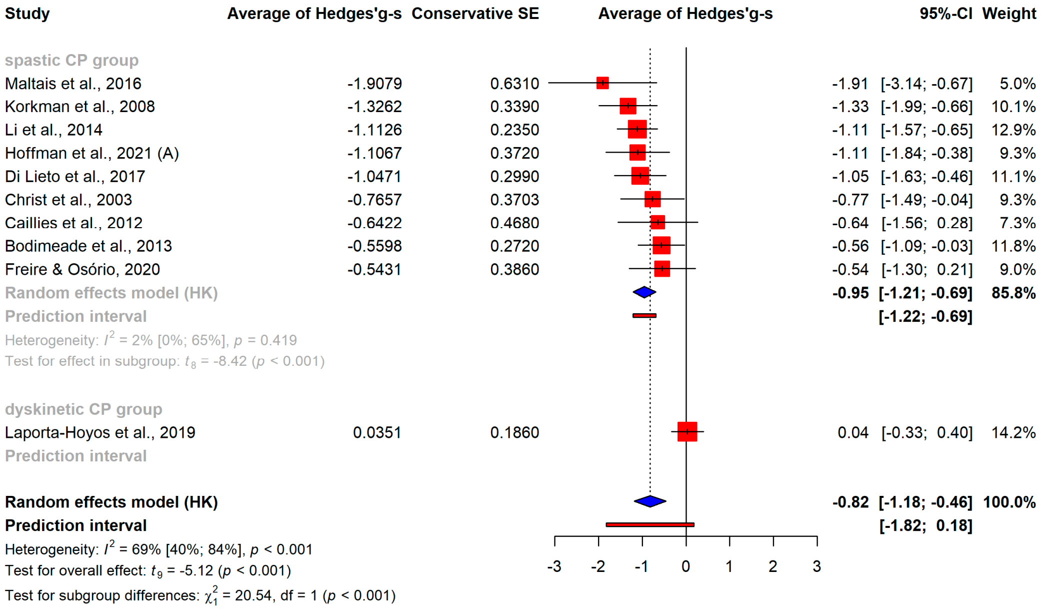

Figure S13. Comparison of effect sizes on inhibitory control by diagnosis.

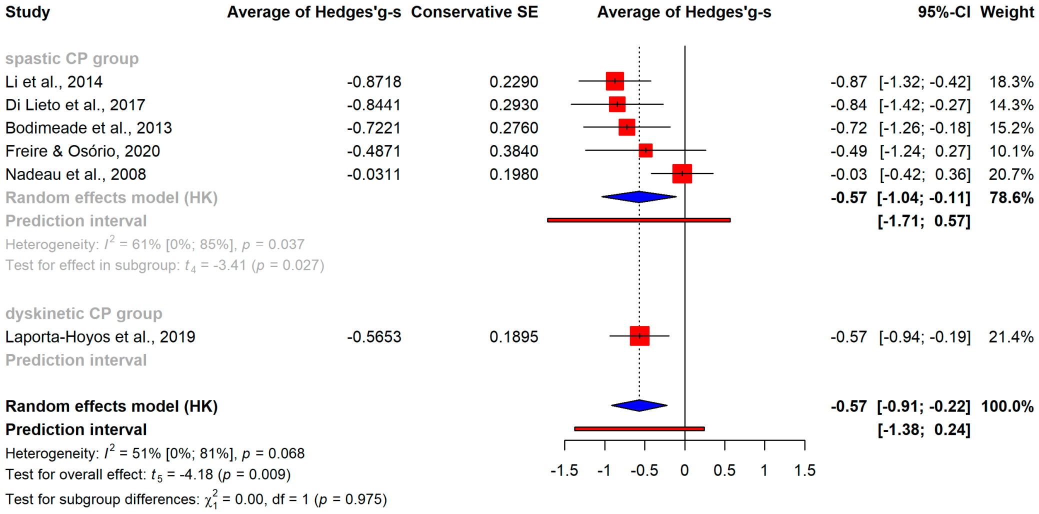

Figure S14. Comparison of effect sizes on flexibility by diagnosis.

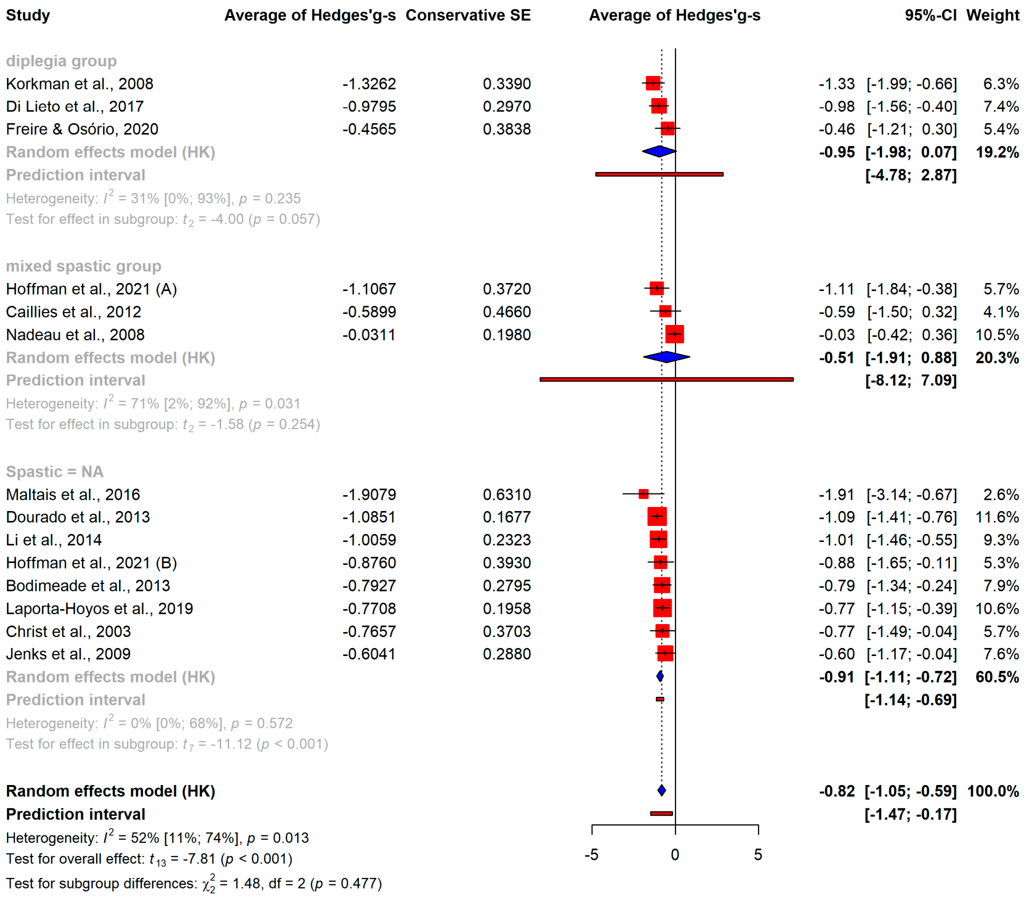

Figure S15. Comparison of effect sizes according to diagnosis.

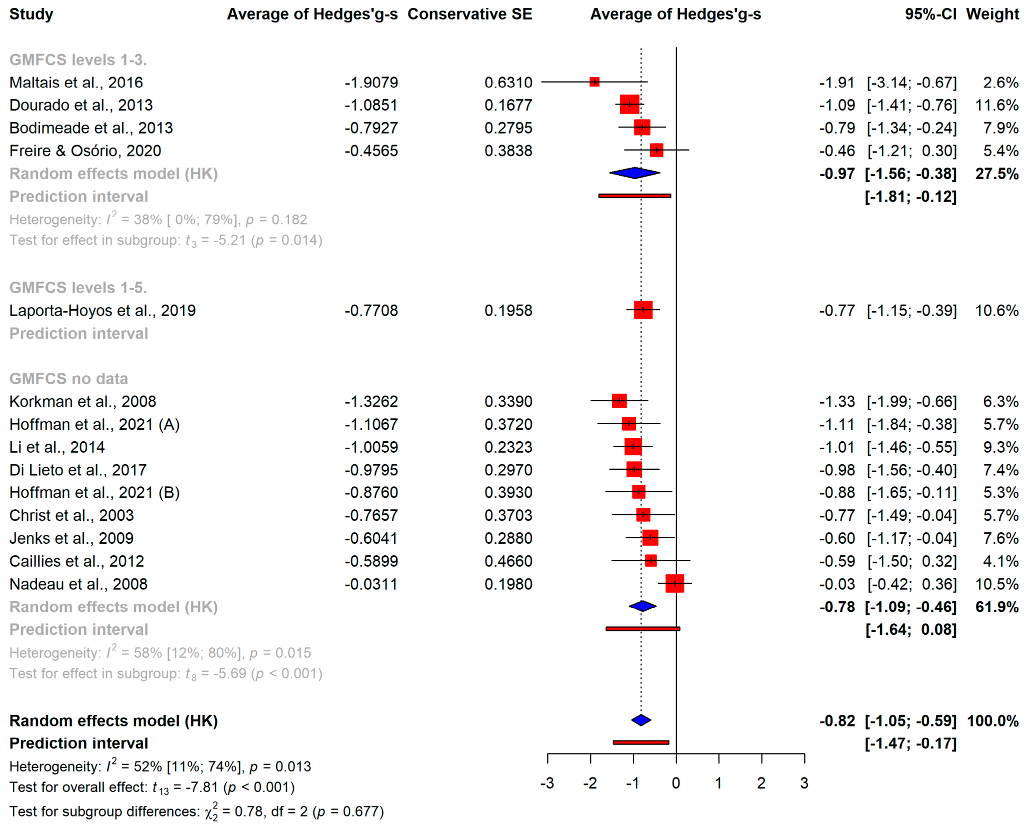

Figure S16. Comparison of effect sizes by GMFCS.

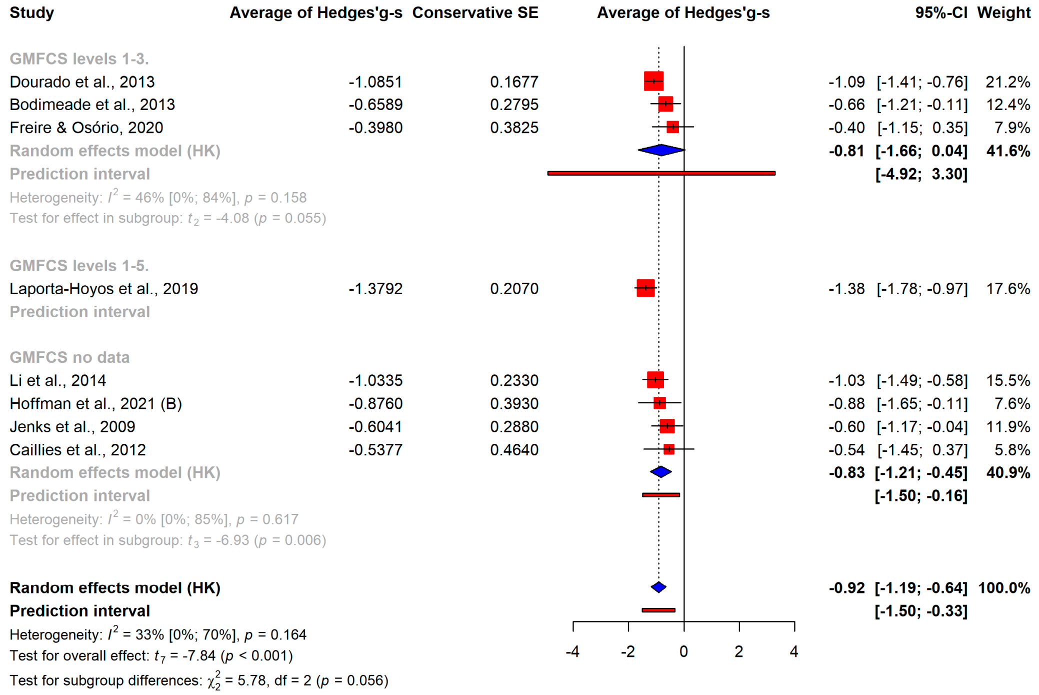

Figure S17. Comparison of effect sizes on working memory by GMFCS.

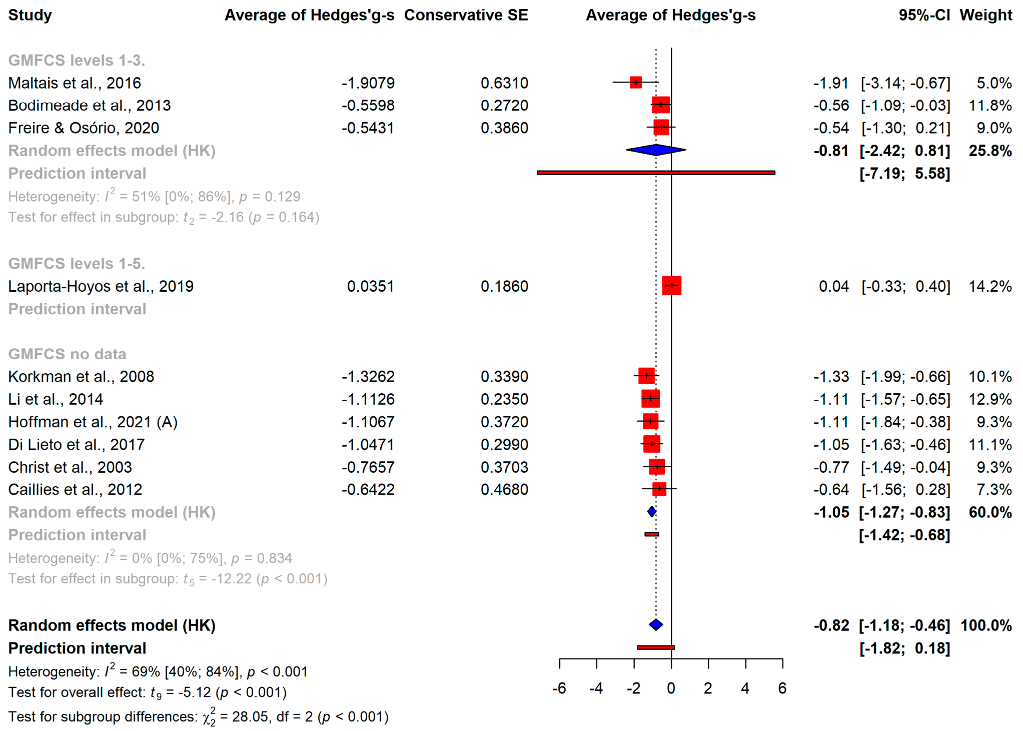

Figure S18. Comparison of effect sizes on inhibitory control by GMFCS.

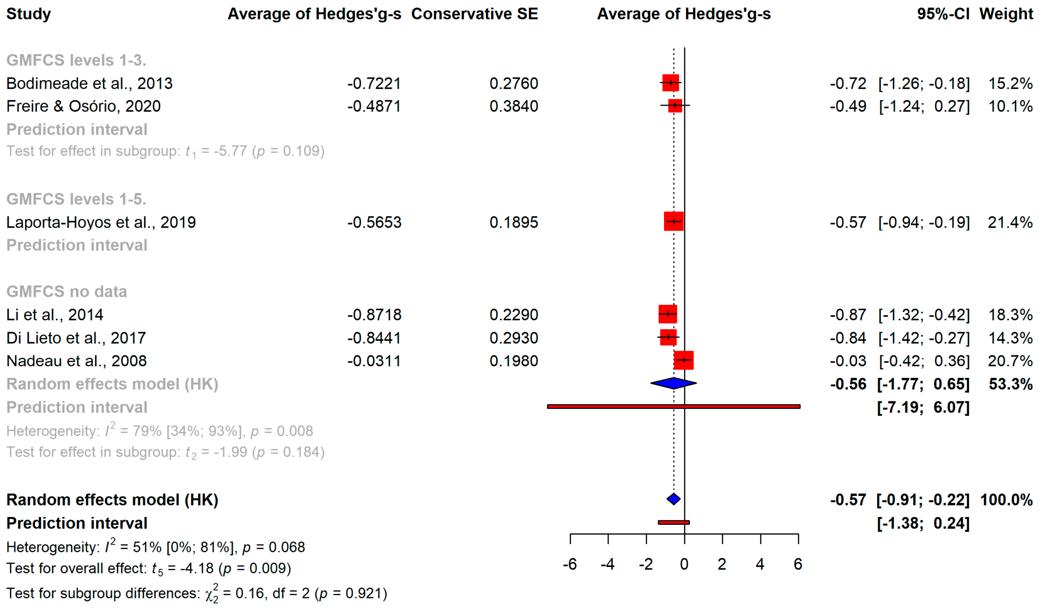

Figure S19. Comparison of effect sizes on flexibility by GMFCS.

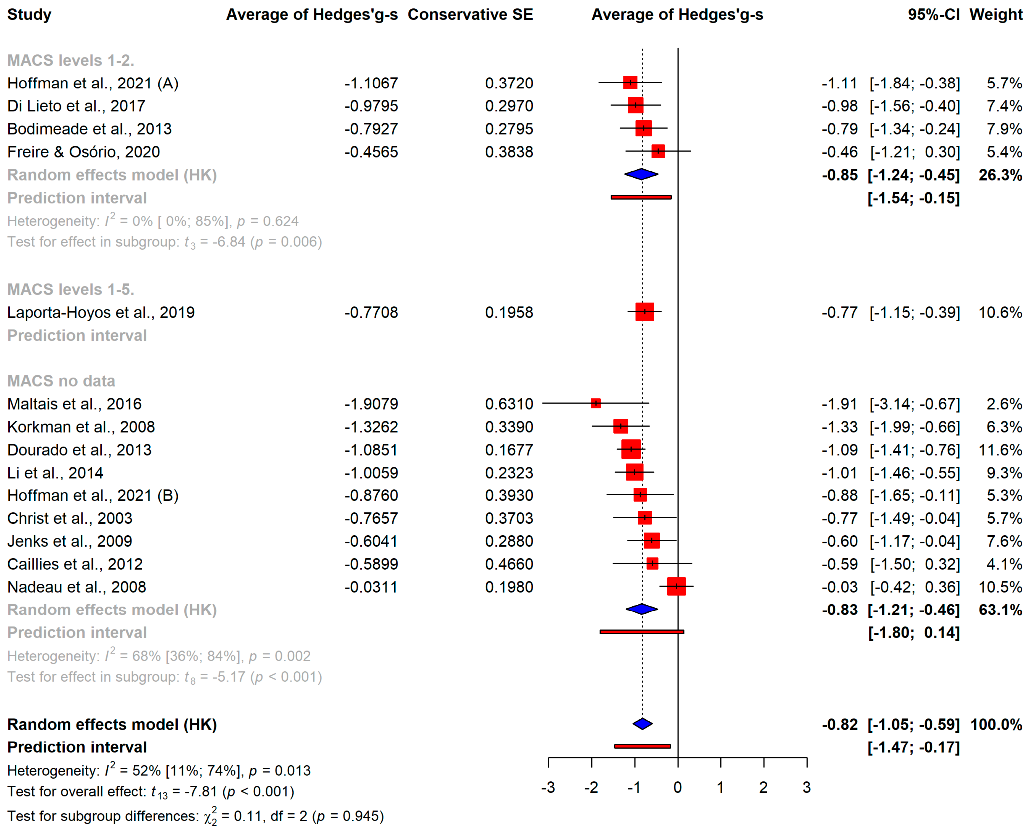

Figure S20. Comparison of effect sizes by MACS.

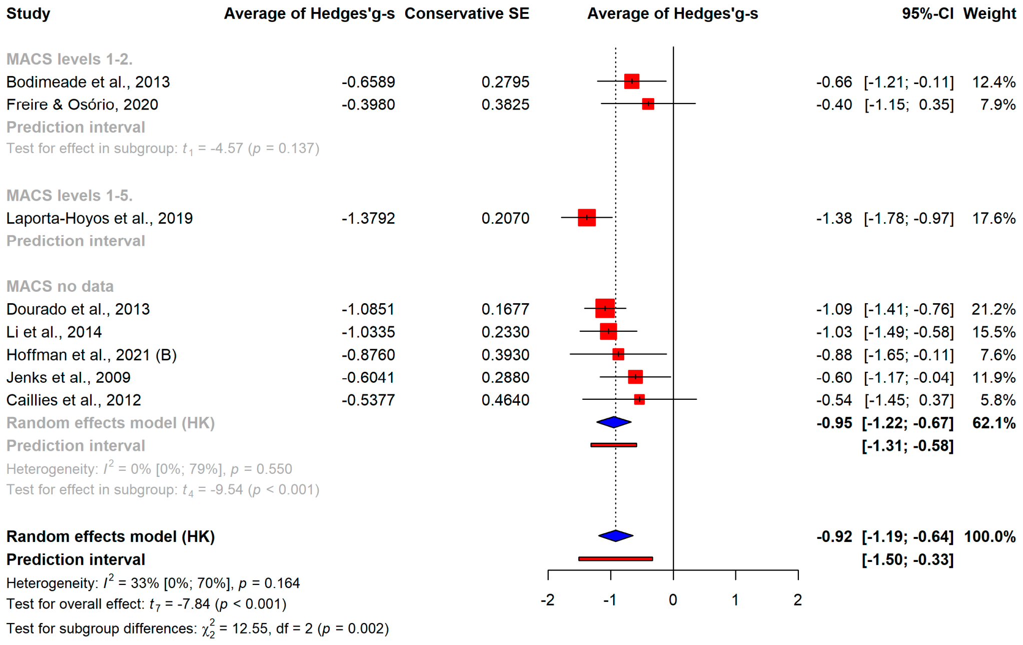

Figure S21. Comparison of effect sizes on working memory by MACS.

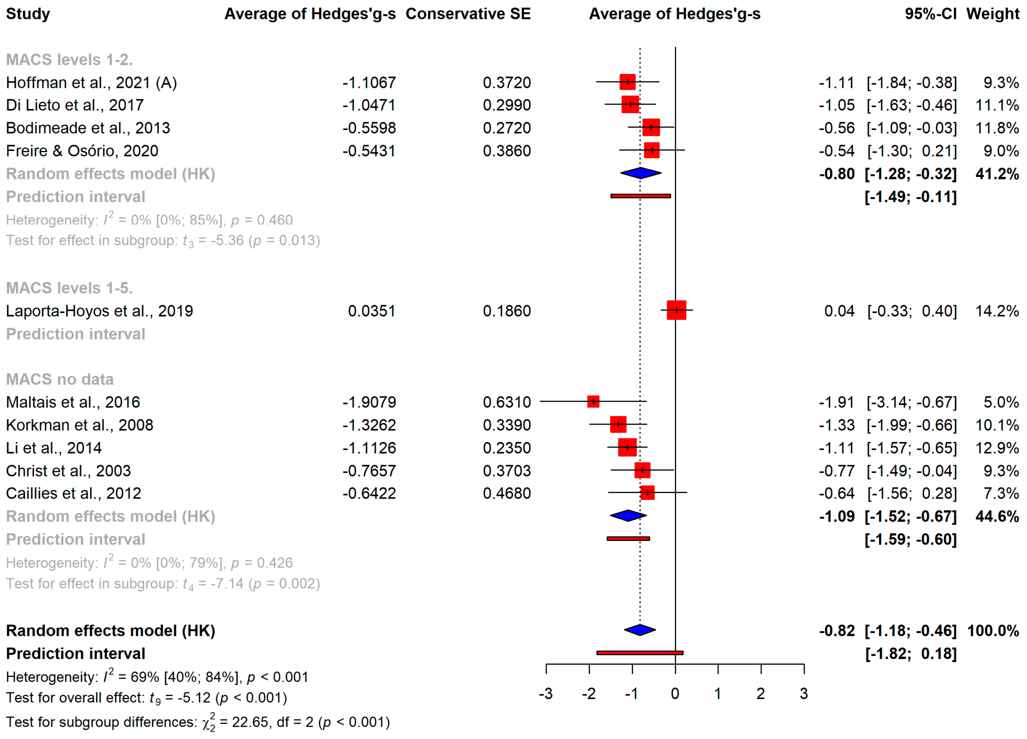

Figure S22. Comparison of effect sizes on inhibitory control by MACS.

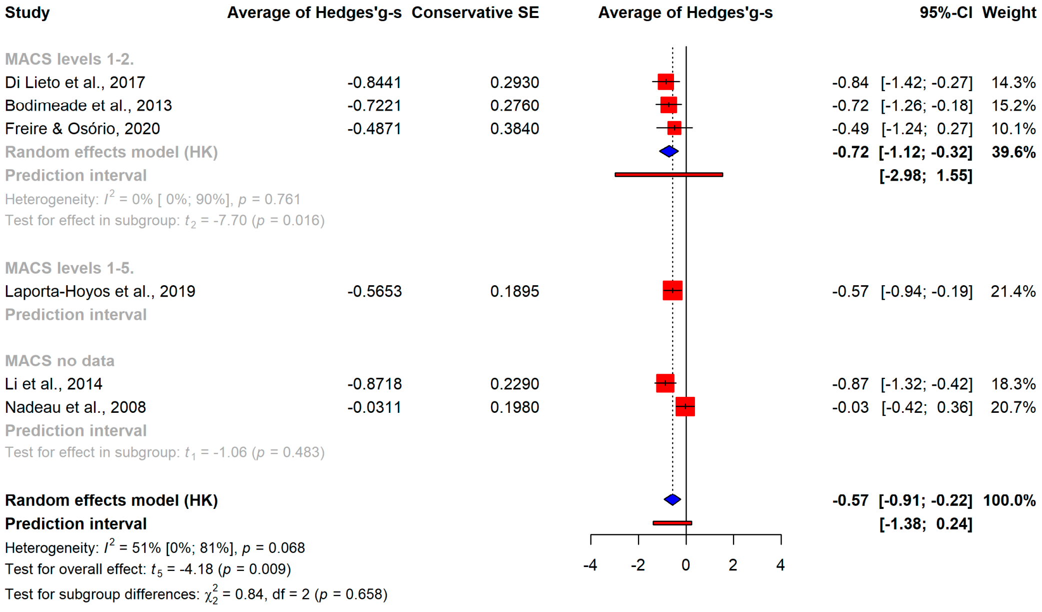

Figure S23. Comparison of effect sizes on flexibility by MACS.

Supplemental S7 - Methodological differences

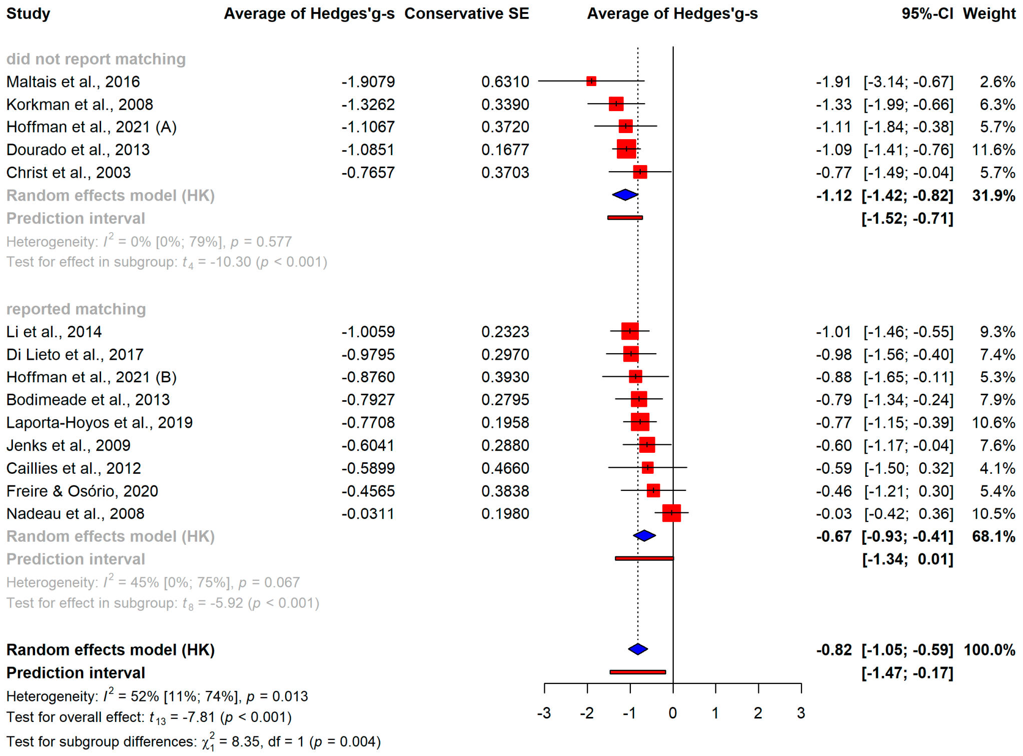

Figure S24. Comparison of effect sizes by matching.

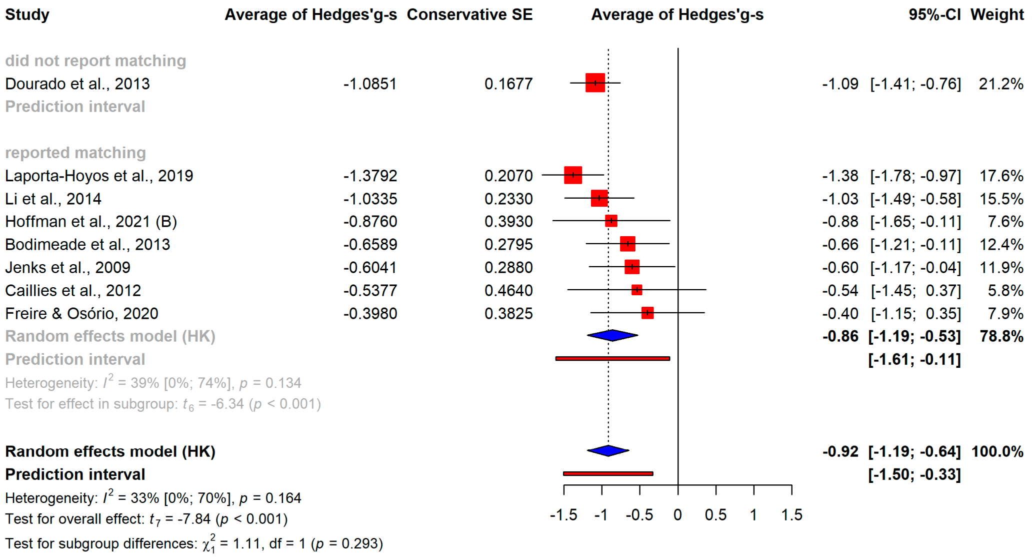

Figure S25. Comparison of effect sizes on working memory by matching of CP and control groups.

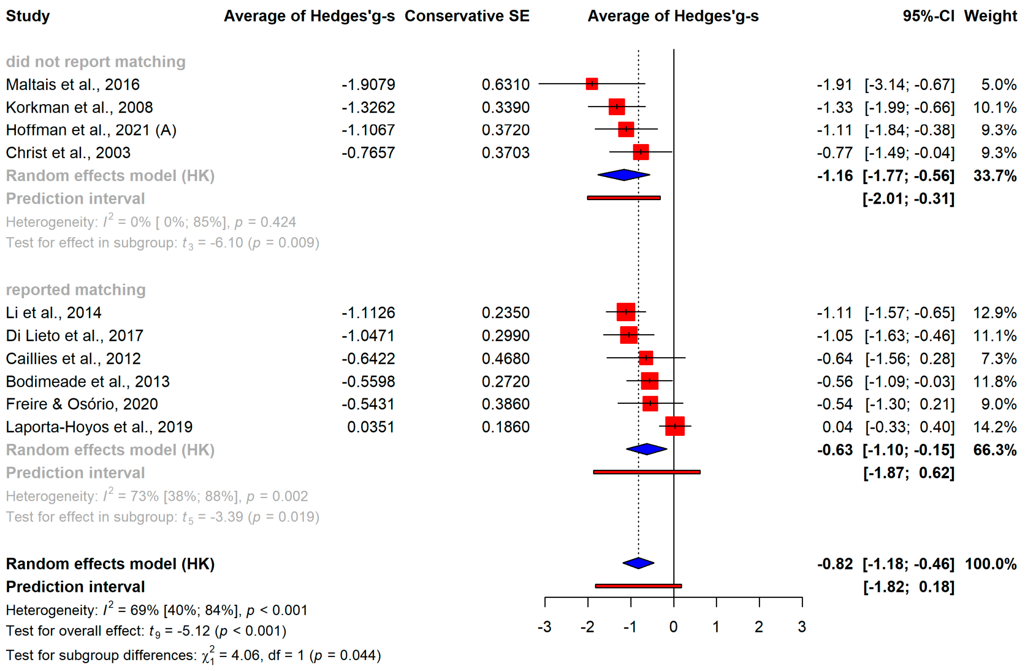

Figure S26. Comparison of effect sizes on inhibitory control by matching of CP and control groups.

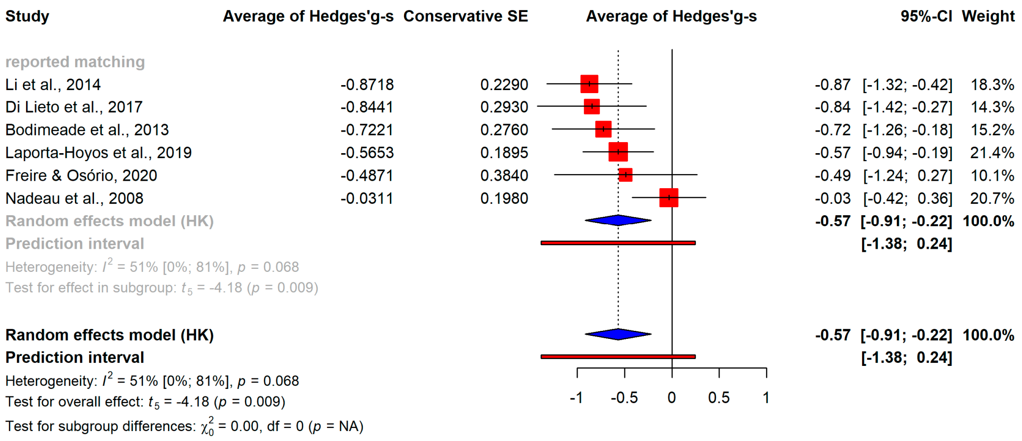

Figure S27. Comparison of effect sizes on flexibility by matching of CP and control groups.

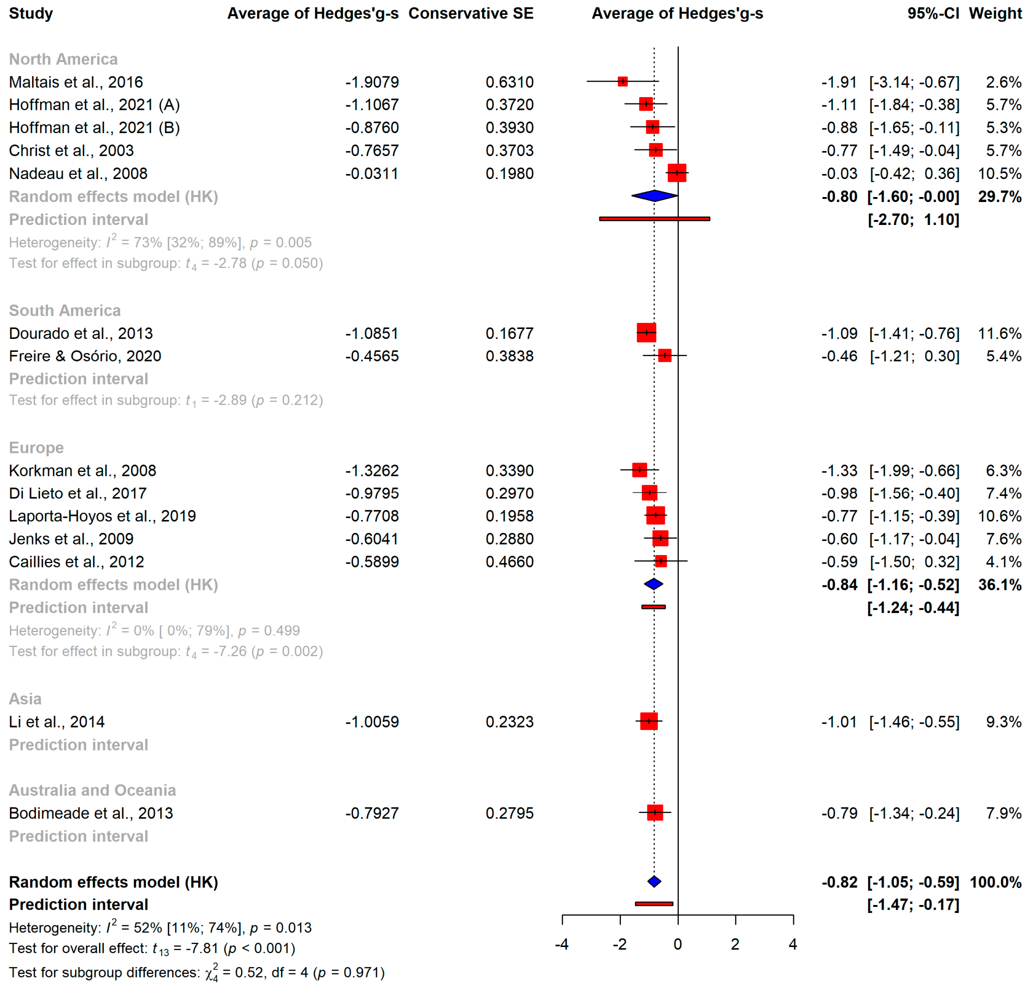

Figure S28. Comparison of effect sizes by continent.

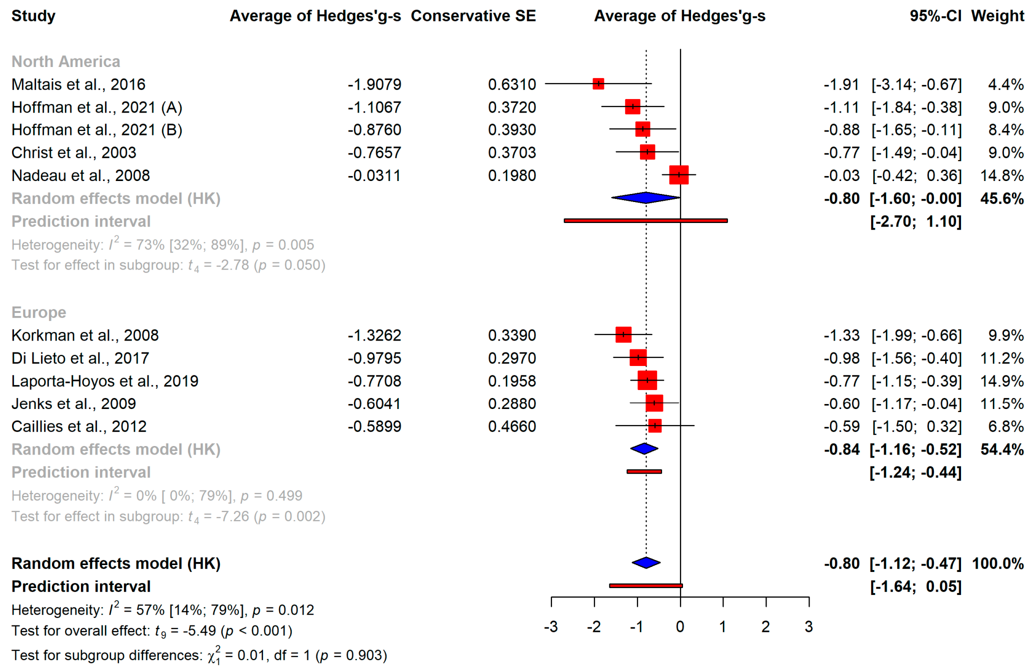

Figure S29. Comparison of effect sizes between North-America and Europe.

Table S1. Results of meta-regression analyses for methodological differences.

Regression	Executive function	Coefficient	*k*	SE	95%CI		*p*
					LB	UB	
Gender distribution (F)	Overall executive function	0.0058	13	0.0120	- 0.0206	0.0322	0.6374
	working memory	0.0083	6	0.0127	- 0.0269	0.0434	0.5501
	inhibitory control	- 1.0023	10	1.7020	- 4.9272	2.9225	0.5722
	flexibility	- 2.5629	6	2.6310	- 9. 8678	4.7420	0.3852
Mean age	Overall executive function	0.0007	15	0.0157	- 0.0332	0.0345	0.9667
	working memory	- 0.0179	8	0.0148	- 0.0542	0.0184	0.2733
	inhibitory control	0.0615	10	0.0275	- 0.0020	0.1249	0.0559
	flexibility	0.0056	6	0.0314	- 0.0817	0.0929	0.8665
Year of publications	Overall executive function	- 0.0174	15	0.0179	- 0.0561	0.0214	0.3503
	working memory	- 0.0317	8	0.0325	- 0.1113	0.0478	0.3664
	inhibitory control	0.0228	10	0.0319	- 0.0507	0.0963	0.4943
	flexibility	- 0.0447	6	0.0300	- 0.1280	0.0385	0.2101

Note: CI = confidence interval, LB =lower bound; UB = upper bound.

Supplemental S8 - Publication Bias

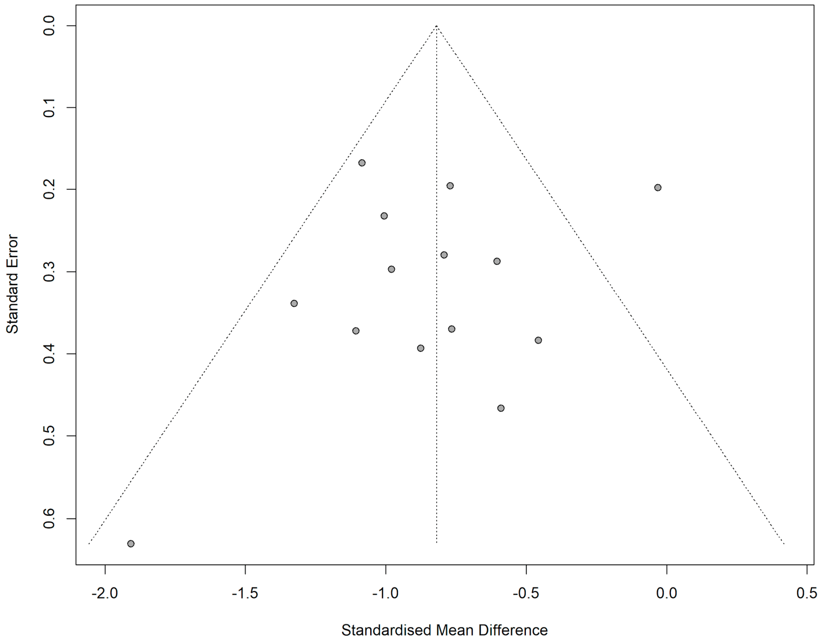

Figure S30. Funnel plot of studies reporting on measure of executive functions.

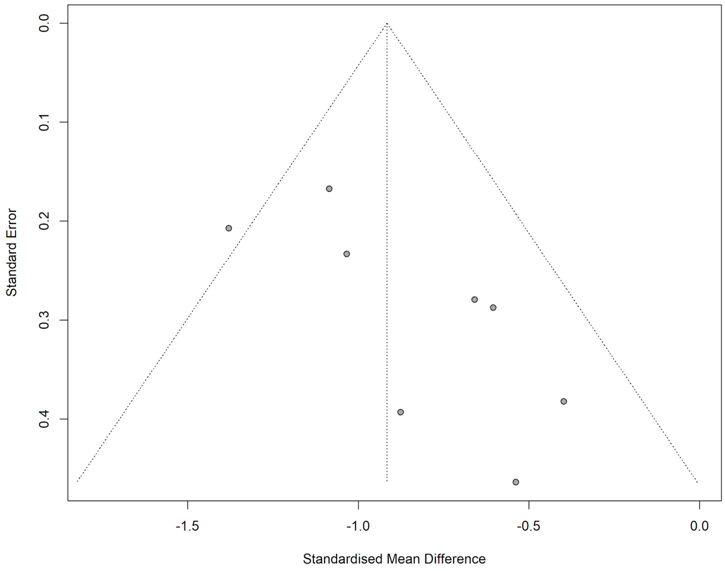

Figure S31. Funnel plot of studies reporting on a measure of working memory.

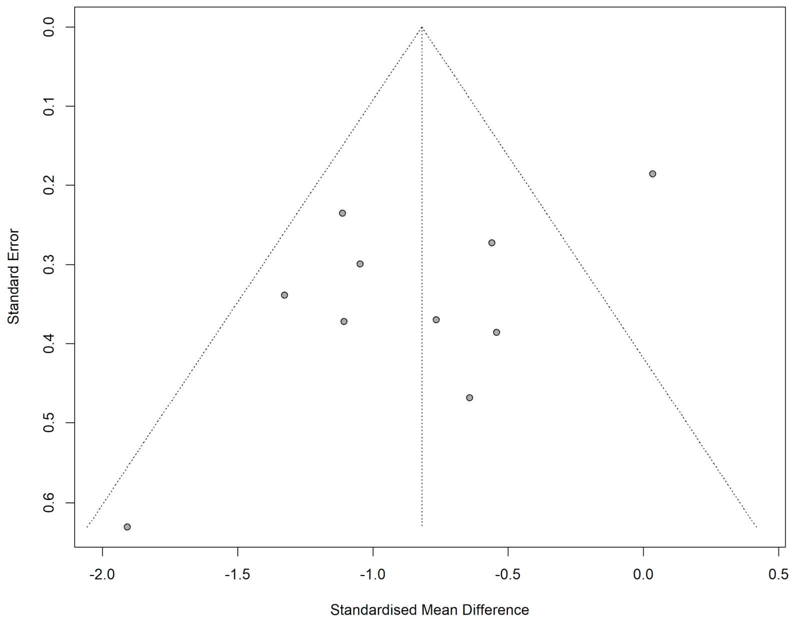

Figure S32. Funnel plot of studies reporting on a measure of inhibitory control.

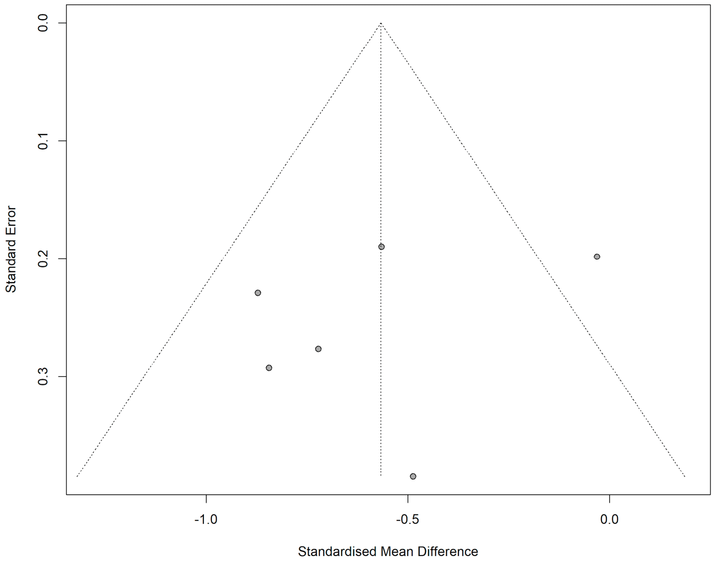

Figure S33. Funnel plot of studies reporting on a measure of flexibility.
